# In silico analysis decodes transthyretin (TTR) binding and thyroid disrupting effects of per- and polyfluoroalkyl substances (PFAS)

**DOI:** 10.1007/s00204-022-03434-8

**Published:** 2022-12-25

**Authors:** Rupal Dharpure, Subrata Pramanik, Ajay Pradhan

**Affiliations:** 1 Jyoti and Bhupat Mehta School of Health Science and Technology, Indian Institute of Technology Guwahati, Guwahati, Assam 781039 India; 2 Biology, The Life Science Center, School of Science and Technology, Örebro University, 70182 Örebro, Sweden

**Keywords:** Thyroid toxicity, Transthyretin, Molecular interactions, H-bond and Hydrophobic interactions, Binding energy, Hypothyroidism, Amyloidogenesis

## Abstract

Transthyretin (TTR) is a homo-tetramer protein involved in the transport of thyroid hormone (thyroxine; T4) in the plasma and cerebrospinal fluid. Many pollutants have been shown to bind to TTR, which could be alarming as disruption in the thyroid hormone system can lead to several physiological problems. It is also indicated that the monomerization of tetramer and destabilization of monomer can lead to amyloidogenesis. Many compounds are identified that can bind to tetramer and stabilize the tetramer leading to the inhibition of amyloid fibril formation. Other compounds are known to bind tetramer and induce amyloid fibril formation. Among the pollutants, per- and polyfluoroalkyl substances (PFAS) are known to disrupt the thyroid hormone system. The molecular mechanisms of thyroid hormone disruption could be diverse, as some are known to bind with thyroid hormone receptors, and others can bind to membrane transporters. Binding to TTR could also be one of the important pathways to alter thyroid signaling. However, the molecular interactions that drive thyroid-disrupting effects of long-chain and short-chain PFASs are not comprehensively understood at the molecular level. In this study, using a computational approach, we show that carbon chain length and functional group in PFASs are structural determinants, in which longer carbon chains of PFASs and sulfur-containing PFASs favor stronger interactions with TTR than their shorter-chained counterparts. Interestingly, short-chain PFAS also showed strong binding capacity, and the interaction energy for some was as close to the longer-chain PFAS. This suggests that short-chain PFASs are not completely safe, and their use and build-up in the environment should be carefully regulated. Of note, TTR homologs analysis suggests that thyroid-disrupting effects of PFASs could be most likely translated to TTR-like proteins and other species.

**Supplementary Information:**

The online version contains supplementary material available at 10.1007/s00204-022-03434-8.

## Introduction

Per- and polyfluoroalkyl substances (PFASs) are a large family of environmentally persistent, man-made fluorinated compounds (Rickard et al. 2022). PFASs have different biological, structural, chemical, and physical properties. For instance, they can differ in molecular polarity, thermal stability, chemical stability, hydrophobicity, lipophilicity, and biological resistance to degradation (Blake et al. 2018; Buck et al. 2011; Kim et al. 2016). These properties lead to PFASs being used in a wide variety of products, such as food packaging, cooking utensils, water- and thermal-resistance materials, non-stick and protective coatings, and firefighting foams (Brendel et al. 2018; Chambers et al. 2021; Lindstrom et al. 2011). As a result, PFASs have been detected in ground and surface water, sediments, blood, and urine (Calafat et al. 2019; Cousins and Vestergren 2011; Han et al. 2018; Wang et al. 2014). Based on their carbon backbone number, PFASs are divided into long-chain (containing ≥ 6 carbons), short-chain (containing 6 < carbons < 3) and ultrashort-chain (containing ≤ 3 carbons) PFAS (Chambers et al. 2021).

Long-chain PFASs have been used for a long time and are known to be resistant to degradation. Studies have shown that long-chain PFASs, including well-known PFOS and PFOA, accumulate and remain in the environment for a long time (Neagu et al. 2021; Zeng et al. 2019). The half-life of PFOS has been determined as 4 and 40 years in human body and in the environment, respectively (Olsen et al. 2017). The main manufacturer of PFOS in the USA, 3 M, phased out its production in 2000 (Ren et al. 2016). Extensive use, production and detection of long-chain PFASs in the environment resulted in production of short-chain PFASs (Kato et al. 2011). Although these alternative plasticizers were proposed to decrease environmental and physiological burdens, they are as persistent as long-chain PFASs and more mobile in aquatic systems. As a result, short-chain PFASs are also being detected in the environment and humans (Brendel et al. 2018; Olsen et al. 2017). Besides, other compounds including perfluorotelomer alcohols have a potential to degrade to PFOA in organisms and subsequently cause humans to be additionally exposed to PFASs (Martin et al. 2005). The data on health risks associated with long- and short-chain PFASs are becoming evident. Several studies have shown that exposure to PFASs may lead to disruption of endocrine and immune systems, neurotoxicity, and defects in fetal development, damage to the liver and kidney, and carcinogenic effects (Lau et al. 2007; Olsen et al. 2017).

Thyroid gland produces thyroid hormones (thyroxine; T4 and triiodothyronine; T3) that regulate metabolism, cellular growth, immune system, reproduction, development, heat production and circadian rhythm (Brent 2012; Ikegami et al. 2019; Klecha et al. 2000; Mullur et al. 2014). T3 and T4 produced by the thyroid gland are transported to plasma by transport protein transthyretin (TTR) (Vieira and Saraiva 2014). TTR is a highly conserved soluble homo-tetramer protein that is found in several vertebrates (Power et al. 2000). TTR is synthetized by the liver and the choroid plexus of brain which are the main sources of TTR in plasma and cerebrospinal fluid (CSF), respectively (Aleshire et al. 1983; Vieira and Saraiva 2014). Along with its role as a carrier protein of thyroid hormones and retinol in plasma and CSF, TTR is also associated with nervous system physiology (Vieira and Saraiva 2014). It has been indicated that differences in TTR levels could be involved in various diseases of the nervous system, including cerebral ischemia and Alzheimer’s disease (Vieira and Saraiva 2014). Altered expression of TTR may also lead to changes in thyroid hormone level that may result in hypothyroidism/hyperthyroidism which in turn causes several health problems, such as altered metabolism, cardiac output, gastrointestinal function, neuropsychiatric and reproductive abnormalities, nervousness and palpitation, weight loss or gain, infertility, paralysis, mental problems, insomnia, etc. (Mounika et al. 2013; Williams 2008). According to American Thyroid Association, 12% of the US population is expected to develop thyroid problem during their lifetime and around 20 million people have some form of thyroid problems (Association 2019).

The increasing levels of environmental pollutants have been implicated in thyroid-related problems (Mughal et al. 2018). It is indicated that PFASs can disrupt the normal functioning of the thyroid system by altering the levels of thyroid hormones, leading to thyroid diseases (Berg et al. 2015; Dallaire et al. 2009; Liu et al. 2011; Lopez-Espinosa et al. 2012; Melzer et al. 2010; Thibodeaux et al. 2003; Webster et al. 2014; Yu et al. 2009). However, there is a lack of data on how PFASs interfere with proteins involved in the thyroid system and alter thyroid hormone levels. Computational approaches, such as molecular docking and molecular dynamics simulations, are powerful tools for determining macromolecular structure-to-function relationships. These tools could help reveal the mode of action of pollutants by predicting interactions between ligand and protein at macromolecular counterparts in full atomic detail (Hollingsworth and Dror 2018; Hospital et al. 2015). Currently, there are limited studies focusing on binding affinities of PFASs to TTR (Kar et al. 2017; Ren et al. 2016; Weiss et al. 2009). In silico studies could enhance our knowledge about the molecular interactions of PFASs with TTR and their structural attributes. The basic information obtained from in silico models could be critical in designing new PFASs and the infrastructure to develop a database for PFAS screening.

In this study, we have used in silico approach to screen long, short- and ultrashort-chain PFASs for their ability to bind human TTR and compared it to the long-chain PFASs. We show that short-chain PFASs could be as toxic as the long-chain PFASs in regulating the thyroid hormone system.

## Materials and methods

### PFAS dataset

A dataset comprising 15 short-chain and 16 long-chain PFASs (*N*_total_ = 31) was selected from literature survey (Supplemental data Table S1) (Almeida et al. 2021; Brown-Leung and Cannon 2022; Kar et al. 2017; Weiss et al. 2009). Their PubChem compound ID (CID) and 3D structures were retrieved from PubChem (https://pubchem.ncbi.nlm.nih.gov) (Kim et al. 2019). Energy minimization of each structure was done by Avagadro software using MMFF94 classical force field (Kim et al. 2016). 3D structures of a few PFASs were unavailable on PubChem and these structures were drawn using YASARA followed by energy minimization in the same software (Krieger and Vriend 2014; Land and Humble 2018). Each 3D structure file was converted into pdbqt format using OpenBabelGUI (version 3.1.1) (O'Boyle et al. 2011).

### Protein preparation

The starting coordinates of human transthyretin (TTR) were taken from the X-ray crystal structure of human transthyretin (PDB ID: 1ICT, resolution 3.00 Å) (Wojtczak et al. 2001). It was prepared using mgltools from AutoDock (version 1.5.7) (Seeliger and de Groot 2010). Water molecules were deleted, polar hydrogens were added, followed by repairing the missing atoms and lastly adding the Kollman charges using mgltools (Bikadi and Hazai 2009; Huey and Morris 2008; Seeliger and de Groot 2010; Umesh et al. 2021). As T4 binds at the interface of the A–C and B–D interfaces of TTR, respectively, we used a dimer of A and C monomers for docking study (Tomar et al. 2012). Cubic grid box was set around the T4 extended to 1 Å, followed by its removal from the binding site for docking of PFASs using mgltools (Bikadi and Hazai 2009; Huey and Morris 2008; Seeliger and de Groot 2010; Umesh et al. 2021). Protein file was saved as pdbqt format.

### Molecular docking of PFASs with TTR and binding energy calculation

Docking of T3, T4, and PFASs with TTR dimer was performed using AutoDock Vina (Seeliger and de Groot 2010). Performance of docking protocol was evaluated by firstly docking the natural ligands T4 and T3 and with TTR and then comparing the docking score with reference (Kar et al. 2017). Output binding energies were noted for each docking. Docked PFAS-TTR complexes were analyzed using LigPlot ^+^- version 2.2.5 software for studying molecular interactions and identifying important interacting amino acids (Laskowski and Swindells 2011). Graphics were prepared using LigPlot ^+^- version 2.2.5 and PyMOL (Janson and Paiardini 2021; Laskowski and Swindells 2011).

### In silico generation of TTR substitutions and docking

Upon identification of the interacting amino acids with PFASs, key interacting amino acids were substituted in the TTR protein with glycine as it does not have an interacting side chain (Kim and Friesner 1997; Prento 2007). Single and multiple amino acid substitutions were performed using YASARA followed by energy minimization of the substituted TTR (Krieger and Vriend 2014; Land and Humble 2018). Docking of PFAS with substituted TTR protein was performed as described in Sect. Molecular docking of PFASs with TTR and binding energy calculation (Seeliger and de Groot 2010). LigPlot ^+^ -version 2.2 by EMBL-EBI was employed to compare interactions of wild-type TTR and substituted TTR with PFAS, respectively (Laskowski and Swindells 2011).

### PFASs interspecies toxicity with TTR

Homologs of human TTR were obtained from NCBI homologous protein database (https://www.ncbi.nlm.nih.gov/homologene). A total of 12 homologs of TTR were found from 12 different vertebrate species, namely *Danio rerio* (Zebrafish), *Xenopus tropicalis* (Clawed frog), *Gallus gallus* (Junglefowl), *Mus musculus* (House mouse), *Rattus norvegicus* (Brown rat), *Bos taurus* (Cattle), *Chlorocebus aethiops* (Grivet), *Macaca fascicularis* (Macaque), *Macaca mulatta* (Rhesus macaque), *Macaca fuscata* (Japanese macaque), *Pongo abelii* (Sumatran orangutan), *Pan troglodytes* (Chimpanzee), and their sequences were retrieved from NCBI BLAST (https://blast.ncbi.nlm.nih.gov/Blast.cgi) (Ladunga 2009). Multiple sequence alignment was performed using EMBL-EBI Clustal Omega (https://www.ebi.ac.uk/Tools/msa/clustalo/) (Sievers and Higgins 2014). In aligned sequences, amino acids and their positions were matched with that of human TTR to see if the same amino acids are present on the same position or not in the other species, and results were visualized in Jalview software (version 2.11.2.4) (Procter et al. 2021).

## Results

### Long-chain PFASs showed higher toxicity propensity with TTR

**Fig. 1:**
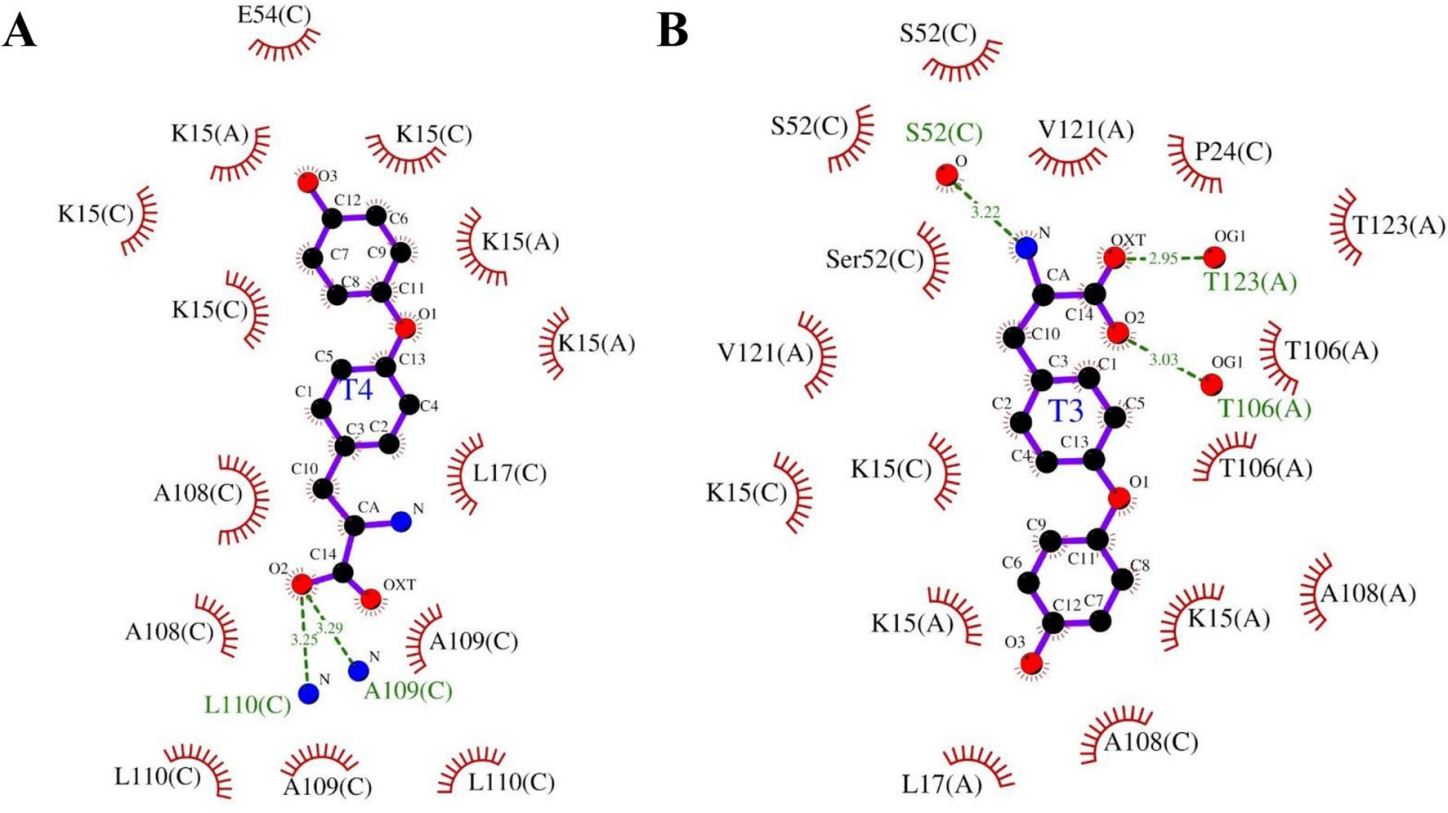
Molecular interactions of natural ligands T4 and T3 with TTR. H-bonds observed between protein and ligand are indicated by green dashed lines along with bond length, while hydrophobic interactions are represented by a brown arc with spokes radiating toward the ligand. Molecular interactions between **A** T4 ( – 6.2 kcal/mol) and **B** T3 ( – 6.2 kcal/mol) with TTR are shown

The toxicity propensity of PFASs towards TTR was evaluated based on their binding energy. In this regard, binding energies of natural ligands T4 and T3 with TTR were found to be – 6.2 kcal/mol as shown in Table S1. At the dimer interface, K15 interacts with T4 and T3, respectively. Additionally, T4 also interacts with L17, E54, A108, A109, and L110 of the chain A of TTR, whereas T3 interacts with L17, T106, A108, V121, and T123 from chain A and P24, S52, and A108 from chain C (Table S1). With respect to molecular interactions, T4 forms H-bonds with L110 and A109 of chain C (amino acid AA of chain A and C are referred to as AA(A) and AA(C), respectively, throughout the text, e.g., L110 of chain C is L110(C)). On the other hand, T3 forms H-bonds with T106(A), S52(C), and T123(A), respectively. Captivatingly, both T3 and T4 formed hydrophobic interactions with various amino acids, e.g., K15(A), K15(C), and L17(C) as shown in Fig. 1 and Table S1.

In respect of PFASs, we calculated binding energy, interacting amino acids, and type of molecular interactions with TTR as explained in Table S2 and Table S3, respectively**.** We first aimed to identify which PFASs have stronger binding affinity with TTR than T4 and T3. To this end, binding energy analysis showed that all the 16 long-chain PFASs exerted stronger binding affinity towards TTR ranging between – 9.8 kcal/mol and – 6.7 kcal/mol (>  – 6.2 kcal/mol) compared to T3 and T4 as shown in Table S1, whereas 10 out of 15 short-chain PFASs showed a stronger binding affinity with TTR compared to T4 and T3 (Tables S1 and S3). We thus next investigated key amino acids K15, A108, A109, L110, S117, T118, and T119 from chain A and K15, A108, L110, S117, and T119 from chain C of TTR interact with long-chain PFASs. Similarly, short-chain PFASs interacted with amino acid L17, A108, A109, L110, S117, T118, and T119 from chain A and A108, A109, L110, S117, T118, and T119 from chain C of TTR (Tables S1–S3). It should the noted that K15 was frequently interacting with long-chain PFASs and not with short-chain PFASs. Besides, residues M13, E54, T106, and V121 interacted less frequently with TTR (Tables S2-S3).

**Fig. 2:**
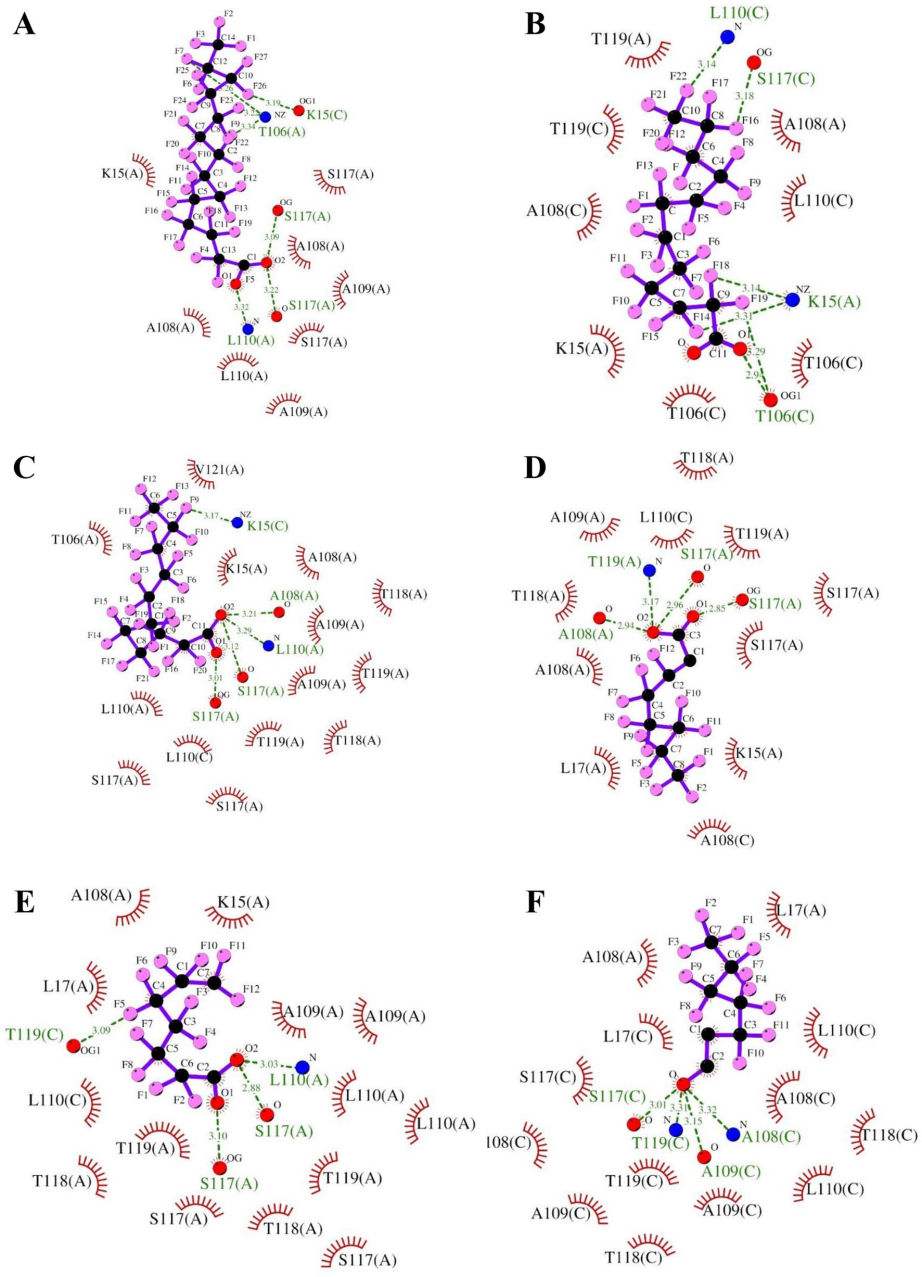
Molecular interactions of long-chain PFASs with TTR. H-bonds observed between TTR and PFASs are indicated by green dashed lines along with bond length, while hydrophobic interactions are represented by brown arcs with spokes radiating toward PFASs. Molecular interactions of **A** perfluorotetradecanoic acid ( – 9.8 kcal/mol), **B** perfluorododecanoic acid ( – 9.4 kcal/mol), **C** perfluoroundecanoic acid ( – 9.3 kcal/mol), **D** 2H-perfluoro-2-octenoic acid ( – 7.4 kcal/mol), **E** 7H-Perfluoroheptanoic acid ( – 7.4 kcal/mol), and **F** 5:2 fluorotelomer alcohol ( – 6.7 kcal/mol) with TTR are shown

**Fig. 3:**
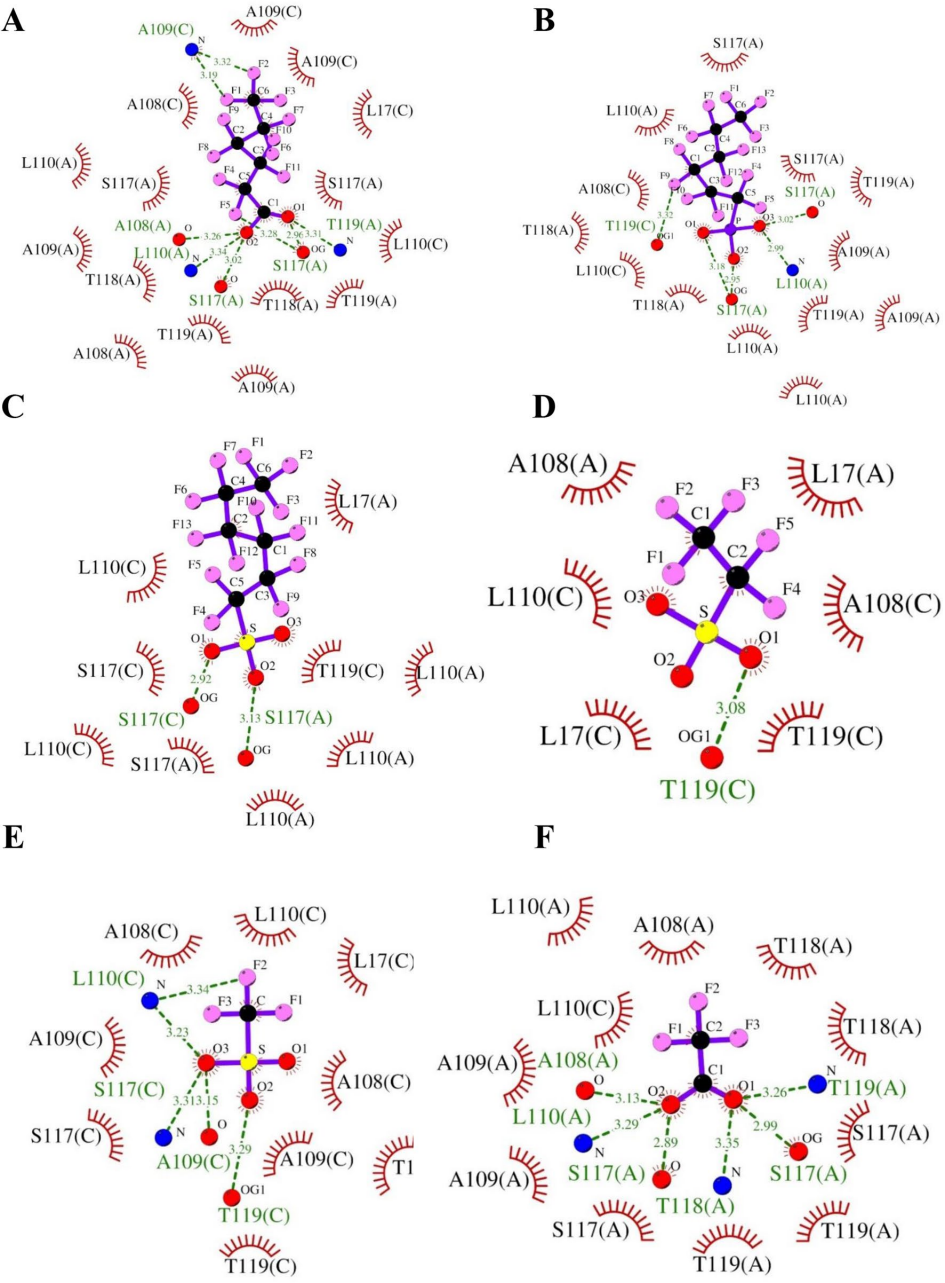
Molecular interactions of short-chain PFASs with TTR. H-bonds observed between protein and ligand are indicated by green dashed lines along with bond length, while hydrophobic interactions are represented by a brown arc with spokes radiating towards ligand. Molecular interactions of **A** perfluorohexanoic acid ( – 7.4 kcal/mol), **B** perfluorohexyl phosphonate ( – 7.3 kcal/mol), **C** perfluorohexane sulfonate ( – 7.3 kcal/mol), **D** perfluoroethane sulfonic acid ( – 4.9 kcal/mol), **E** trifluoromethane sulfonic acid ( – 4.2 kcal/mol), and **F** trifluoroacetic acid ( – 4.0 kcal/mol) with TTR are shown

Regarding the molecular interactions, we observed that H-bonds and hydrophobic interactions drive PFASs binding with TTR. Hydrophobic interactions were major contributors to the molecular interactions, followed by H-bonds (Fig. 2 and Tables S2-S3). PFASs having binding affinity stronger than T4 and T3 ( – 6.2 kcal/mol) form H-bonds predominantly with K15(A), K15(C), T106(A), T106(C), A109(C), L110(A), L110(C), S117(A), S117(C), T118(A), T118(C), T119(A), and T119(C), whereas they form hydrophobic interactions with K15(A), T106(A), T106(C), A108(A), A108(C), A109(A), L110(A), L110(C), S117(A), S117(C), T118(A), T118(C), T119(A), and T119(C) (Tables S2–S3 and Figs. 2 and 3). For example, as shown in Fig. 2, long-chain PFASs, e.g., perfluorotetradecanoic acid ( – 9.8 kcal/mol), perfluorododecanoic acid ( – 9.4 kcal/mol), perfluoroundecanoic acid ( – 9.3 kcal/mol), perfluorotributyl amine (ptba) ( – 9.2 kcal/mol), and perfluorodecane sulfonic acid ( – 9.1 kcal/mol), perfluorooctanoic acid ( – 8 kcal/mol), N-methyl perfluorooctanesulfonamido ethanol ( – 7.6 kcal/mol), and 2H-Perfluoro-2-octenoic acid ( – 7.4 kcal/mol) having stronger interactions with TTR than T4 and T3 form H-bonds with K15(C) and S117(A). Similarly, they form hydrophobic interaction through S117(A), A108(A), and A109(A) as described in Fig. 2.

In the case of short-chain PFASs, when we analyzed binding energy, we found a similar stronger binding affinity (i.e., H-bonds and hydrophobic interactions) like long-chain PFASs for 10 out of 15 short-chain PFASs as elucidated in Fig. 3A–C. For example, perfluorohexanoic acid ( – 7.4 kcal/mol), perfluorohexyl phosphonate ( – 7.3 kcal/mol), and perfluorohexane sulfonate ( – 7.3 kcal/mol) form H-bonds with L110(C) and S117(C) as indicated in Fig. 3A–C. On the other hand, five short-chain PFASs had shown a weaker binding affinity towards TTR compared to natural ligands T4 and T3 (<  – 6.2 kcal/mol). They had chain lengths varying between 1-C and 4-C atoms (Fig. 3D–F and Figure S7). Important interacting amino acids were found to be L17, A108, A109, L110, and T119, whereas T118 and S117 were less frequently involved. For example, perfluoroethane sulfonic acid ( – 4.9 kcal/mol), trifluoromethane sulfonic acid ( – 4.2 kcal/mol), and trifluoroacetic acid ( – 4.0 kcal/mol) formed H-bonds with T119(A) and hydrophobic interactions with L17(A), L17(C), A108(A), A108(C), A109(A), A109(C), L110(A), L110(C), S117(A), S117(C), T118(A), T119(A), and T119(C) (Fig. 3D–F and Table S3).

PFASs having a stronger binding affinity towards TTR than T4 and T3 have the potential to displace natural ligands (e.g., thyroid hormones, T4 and T3) from the binding site of TTR. As apparent from Fig. 2 and Figures S1–S6, these PFASs have carbon atoms ranging between C6 to C15, with occasional C4 to C5, whereas PFASs having a weaker binding affinity towards TTR have carbon atoms of length C1 to C4 (Fig. 3D–F and Figure S7). In summary, it can be suggested that the more the length of a chain of the PFAS, the stronger will be its binding affinity towards TTR.

### K15, L110, and S117 are key interacting residues with TTR

To get a clearer picture of molecular interactions, we sought to detect key interacting residues with TTR. Comparative analysis of data revealed that K15, A108, A109, L110, and S117 are the key interacting amino acid residues of TTR that were involved in the majority of molecular interactions with PFAS (Table S4). Detailed analyses are shown in Figs. 2 and 3, Figures S1–S11, and Table S4. These residues are involved in the formation of H-bonds and hydrophobic interactions.

### Multiple amino acids’ substitution confirms the key interacting amino acid of TTR for its interaction with PFASs

We next inquired whether the identified amino acid substitution with glycine alters the binding efficiency of PFASs with TTR. Critical amino acid residues from TTR involved in interacting with PFASs have been identified as mentioned before (Table S4). To understand the role of each key residue, we generated protein variants of TTR using in silico substitution of amino acid residue with glycine, including two single amino acid variants (K15G and L110G), one double amino acid variant (K15G/L110G), and one multiple amino acids variant (K15G/L110G/S117G). We then directly tested whether these substitutions in TTR could account for the resulting binding energy changes with identified six strongest and six weakest PFASs, respectively (Tables S5-S16). According to the binding energy results, the binding affinity of the selected PFASs was significantly reduced due to substitution compared to wild-type TTR as described in Table 1. Same phenomena were observed for the total number of hydrophobic interactions and H-bonds interactions, which decreased significantly (Tables S5-S16). Together, these results reveal both binding affinity and molecular interactions reduced markedly in long-chain PFASs. In contrast, the reduction for short-chain PFASs was not as marked as long-chain PFASs (Table 1).

**Table 1:** Effect of amino acid substitutions on binding energies of long- and short-chain PFASs with TTR, respectively

(A) Long-chain PFASs		Binding energy with wild-type TTR and variants (kcal/mol)				
		Wild-type	K15G	L110G	K15G/L110G	K15G/L110G/S117G
1	Perfluorotetradecanoic acid	– 9.8	– 8.8	– 8.8	– 8.2	– 7.9
2	Perfluorododecanoic acid	– 9.4	– 8.1	– 8.1	– 7.8	– 7.4
3	Perfluoroundecanoic acid	– 9.3	– 8.4	– 8.6	– 7.4	– 7.2
4	2H -Perfluoro-2-octenoic acid	– 7.4	– 7.1	– 7	– 6.4	– 6.4
5	7H-Perfluoroheptanoic acid	– 7.4	– 6.9	– 6.5	– 6.5	– 6.0
6	5:2Fluorotelomer alcohol	– 6.7	– 6.5	– 6.1	– 5.6	– 5.6

(B) Short-chain PFASs		Wild-type	K15G	L110G	K15G/L110G	K15G/L110G/S117G
1	Perfluorohexanoic acid	– 7.4	– 7.0	– 6.3	– 6.4	– 6.1
2	Perfluorohexyl phosphonate	– 7.3	– 6.8	– 7.2	– 6.5	– 6.3
3	Perfluorohexane sulfonate	– 7.3	– 7.5	– 6.8	– 6.4	– 6.2
4	Perfluoroethane sulfonic acid	– 4.9	– 5.2	– 4.8	– 4.7	– 4.5
5	Trifluoromethane sulfonic acid	– 4.2	– 4.4	– 4	– 4	– 3.9
6	Trifluoroacetic acid	– 4.0	– 4.1	– 3.7	– 3.6	– 3.4

Furthermore, single variants, K15G and L110G, were less effective in reducing the binding affinity and reducing molecular interactions as compared to double amino acid substituted variants (K15G/L110G), whereas multiple amino acid-substituted variant (K15G/L110G/S117G) has been observed to be most effective in reducing binding affinity for both long-chain and short-chain PFASs. Of note, there was a slight increase in the binding affinity of three short-chain PFASs (perfluoroethane sulfonic acid, trifluoromethane sulfonic acid, and trifluoroacetic acid) and one long-chain PFAS (N-methyl perfluorooctane sulfonamidoethanol) due to K15G and L110G substitutions, respectively. Strikingly, there are three sulfur-containing PFASs, i.e., perfluoroethane sulfonic acid, trifluoromethane sulfonic acid, and N-methylperfluorooctanesulfonamidoethanol, which showed slight increase in the binding affinity due glycine substitution. Although its structural determinant remains to be investigated, from this observation, it can be suggested that sulfur containing PFASs are more resistant and hence more toxic (Kwiatkowski et al. 2020).

### TTR homologous analysis predicts PFAS toxicity in other animals

It would be extremely interesting to find out whether the toxicity of PFASs toward human TTR can be transferred to predict and access their toxicity in other animals. To probe that, we detected that TTR is a highly conserved protein during evolution among vertebrates (Prapunpoj and Leelawatwattana 2009). It is a major thyroid hormone-binding protein in vertebrates. Apart from thyroid hormone transport, TTR has several other functions (e.g., neuroprotection and promotion of neurite outgrowth in response to injury) that are the result of evolutionary changes (Hennebry et al. 2006; Liz et al. 2020). Multiple sequence alignment revealed that its primary protein structure in different vertebrates is highly conserved as can be observed in Figure S12. The amino acid residues that are identical at a particular position as of human TTR are denoted by an asterisk (*). The amino acid residue changes between different species are mainly located in the N-terminal terminal and not the binding sites of the TTR (Prapunpoj and Leelawatwattana 2009).

**Fig. 4:**
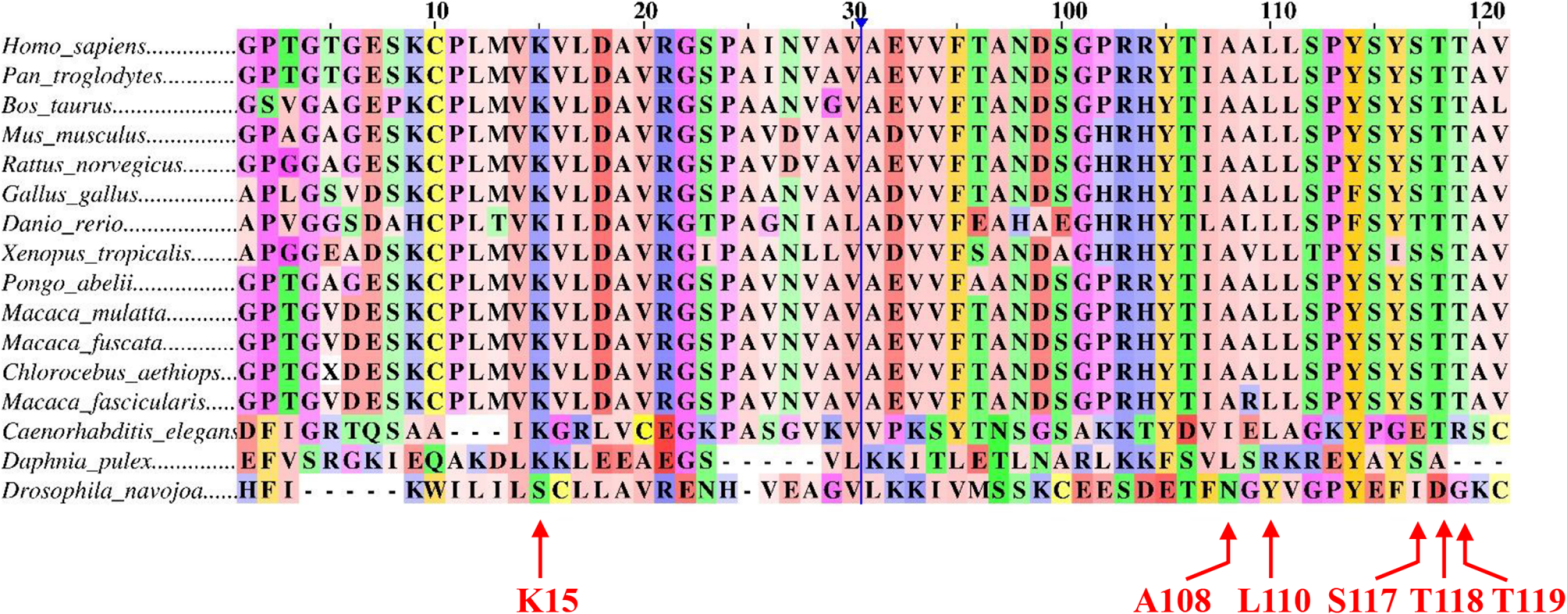
Interspecies toxicity analysis of PFASs in 13 test species using multiple sequence alignment. Interacting residues, including K15, A108, A109, L110, S117, T118, and T119 with PFASs are conserved among different species. Column number 31 to 89 have been hidden for easy visualization of figure and shown by a vertical blue line

It can be seen in Fig. 4 that identified key residues in human TTR, e.g., K15, A108, A109, L110, S117, and T119 for interacting with PFASs are the same amino acids at the same position in all the other vertebrates species, i.e., *Danio rerio* (Zebrafish), *Xenopus tropicalis* (Clawed frog), *Gallus gallus* (Junglefowl), *Mus musculus* (House mouse), *Rattus norvegicus* (Brown rat), *Bos taurus* (Cattle), *Chlorocebus aethiops* (Grivet), *Macaca fascicularis* (Macaque), *Macaca mulatta* (Rhesus macaque), *Macaca fuscata* (Japanese macaque), *Pongo abelii* (Sumatran orangutan), and *Pan troglodytes* (Chimpanzee). Besides, we also found that invertebrates, including *Caenorhabditis elegans* (roundworms), *Daphnia pulex* (Daphnia), and *Drosophila navojoa* (flies) are enriched with TTR orthologs. It can be noted that A108I, S117E, and T119R substitutions were observed in *Caenorhabditis elegans* (Lipton et al. 2004). Likewise, A108L, L110R, and T118A substitutions were observed in *Daphnia pulex* (Kim et al. 2015)*.* In contrast, *Drosophila navojoa* exhibits substitutions in these positions as indicated in Fig. 4 (Kockel et al. 2001; Vanderlinde et al. 2019). In summary, this observation indicates that PFASs are capable of interacting with TTR and TTR-like proteins in other animal species in a similar mechanism as they interact with human TTR.

## Discussion

**Fig. 5:**
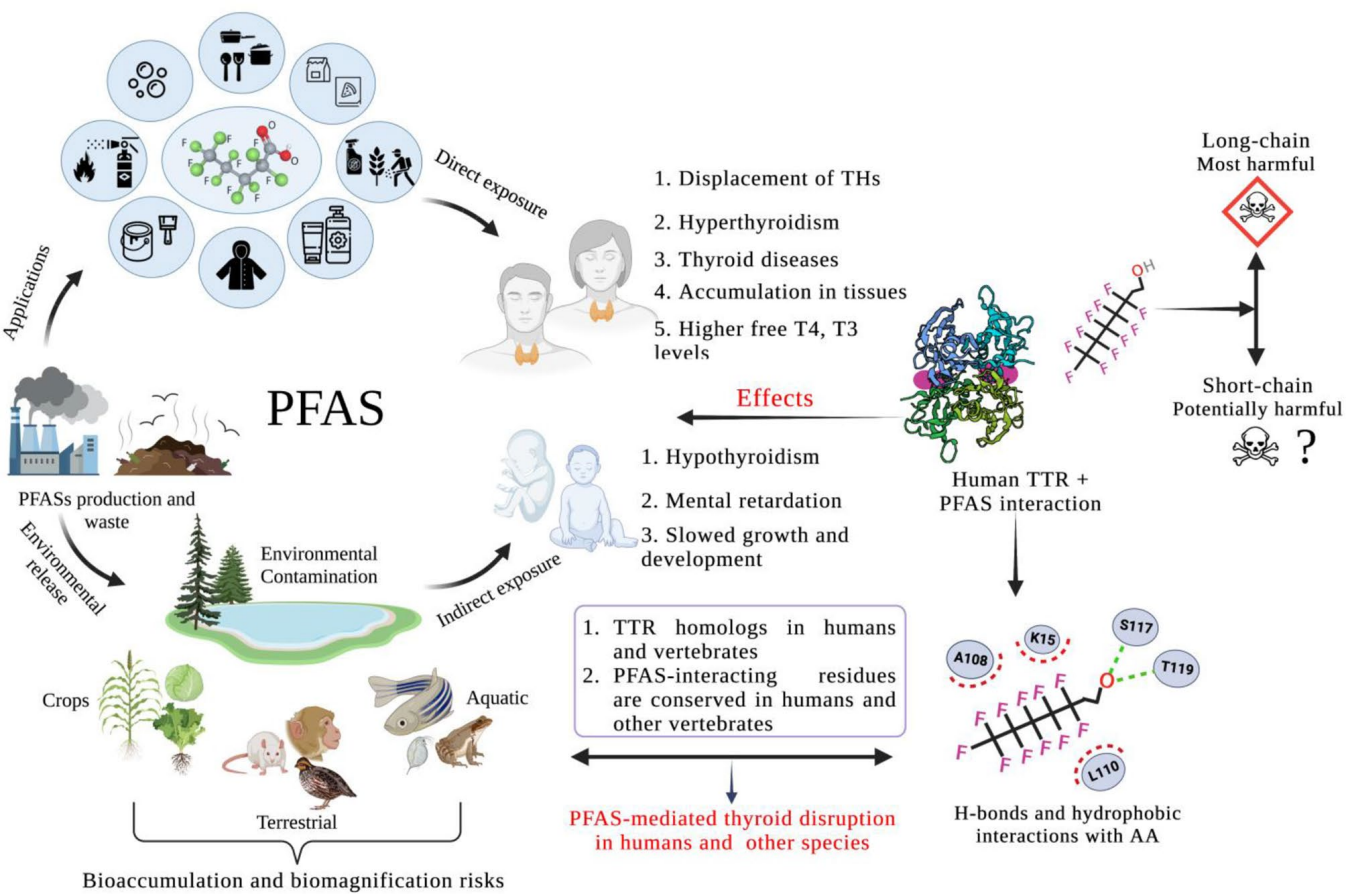
PFAS toxicity in humans and other vertebrates. PFASs enter the ecosystem during their manufacturing and disposal. Humans get exposed to PFASs either through direct exposure from products or indirect environmental exposure. Environmental contamination results in toxicity threats to the biota. Insight into PFAS and human TTR interaction revealed that long and short-chain PFASs are capable of exerting toxicity by displacing THs and bonding with amino acid residues in the form of H-bonds and hydrophobic interactions. The similarity among residues and their position between human TTR and its homologs from other vertebrates indicates that the PFASs toxicity can be translated to other vertebrates and likely other species. (*THs* Thyroid hormones, *T4* Thyroxine, *T3* Triiodothyronine, *PFAS* per- and polyfluoroalkyl substances, *TTR* Transthyretin, *AA* Amino acid). The figure was generated using Biorender software

A molecular and biochemical understanding of PFASs toxicity is of great importance for toxicity assessment, improving public health, environmental pollution management, and sustainable management of ecosystems (Camdzic et al. 2022; Fenton et al. 2021; Frumkin 2001; Lehman-McKeeman and Armstrong 2022; Rericha et al. 2021). Therefore, a general molecular understanding of the structural and molecular determinants that drive PFAS toxicity will help to re-design eco-friendlier and more suitable PFASs for various applications on the one hand and more effective regulation and mitigation of toxicity on the other. In our study, we examined how long- and short-chain PFASs interact with human TTR. We identified key interacting residues and interactions that drive PFASs binding with TTR and predicted PFAS toxicity in other animals. TTR is a homo-tetrameric carrier protein, which transports thyroid hormones in the plasma and cerebrospinal fluid (Rabah et al. 2019; Richardson et al. 2015). It is also involved in the transport of retinol (vitamin A) in the plasma by associating with retinol-binding protein (RBP) (Raghu and Sivakumar 2004). Besides the carrier role of TTR, many other functions have emerged including proteolysis, nerve regeneration, autophagy, and glucose homeostasis. Behavior, cognition, neuropeptide amidation, neurogenesis, nerve regeneration, and axonal growth are some of the cellular processes where TTR has an important role (Vieira and Saraiva 2014). Further, mutations in the *TTR* gene are implicated in the etiology of several diseases, including amyloidotic polyneuropathy, euthyroid hyperthyroxinaemia, amyloidotic vitreous opacities, cardiomyopathy, oculoleptomeningeal amyloidosis, meningocerebrovascular amyloidosis, and carpal tunnel syndrome (Bekircan-Kurt et al. 2022; Coniglio et al. 2022; Parcha et al. 2022; Takahashi et al. 2022; Yee et al. 2019). Besides, maternal exposure to PFASs can result in serum concentration of PFASs in fetuses and newborns, which leads to hypothyroidism, slow growth and development, and mental retardation as explained in Fig. 5 (Fenton et al. 2021).

It is commonly known that by binding with TTR, PFASs disrupt thyroid activity and functions in both humans and experimental animals (Chain et al. 2020; Coperchini et al. 2021). Their preferential binding to proteins like TTR has been proven to cause abnormal thyroid hormone levels and thyroid diseases in monkeys, rodents, and the general human population (Cui et al. 2020; Power et al. 2000). Along with altering thyroid hormone levels, they have also been found to alter thyroid peroxidase (TPO) enzyme activity in vitro (Fenton et al. 2021). TPO plays a critical role in the generation of thyroid hormones (Godlewska and Banga 2019). However, the molecular mechanism behind the interaction of PFASs with TTR and its resultant toxicity is not comprehensively understood at the molecular level. In our study, we decode the thyroid-disrupting effects of PFASs by understanding molecular interactions between TTR protein and PFASs (Fig. 5).

According to our central hypothesis of this article, both short-chain and long-chain PFASs could be toxic, whereas long-chain PFASs are generally more toxic than short-chain analogs which need to be taken into account. 16 long-chain PFAS were analyzed for their binding affinity towards TTR and all of them had stronger binding affinity than the natural ligands, whereas out of 15 short-chain PFASs, 10 compounds were found to have stronger binding affinity than the natural ligands. It suggests that the PFASs having a stronger binding affinity towards TTR have the potential to displace natural ligands (thyroid hormones) from binding sites of TTR and cause toxicity. Besides, to strengthen our data, we compared the binding affinity of 11 Long-chain PFASs and 4 Short-chain PFASs with TTR and PPARγ (peroxisome proliferator-activated receptor gamma) as previously described (Almeida et al. 2021) (Table S17). For example, the comparison showed that perfluorododecanoic acid did not bind with PPARγ (3.5 kcal/mol), whereas it strongly binds with TTR ( – 9.4 kcal/mol). In contrast, other PFASs, such as perfluorodecane sulfonic acid and perfluorooctane sulfonic acid showed similar binding affinity toward PPARγ and TTR, respectively, as described in Table S17. Likewise, PFASs such as perfluorobutane sulfonic acid, perfluoropentanoic acid, and perfluorobutanoic acid showed a higher binding affinity with PPARγ than TTR. Taken together, these observations indicate the binding specificity of PFASs with different proteins.

With respect to the molecular interactions that drive PFASs binding with TTR, hydrophobic interactions were major contributors to binding energy in PFASs having stronger affinity, whereas H-bonds were the vital interactions in PFASs having weaker binding affinity (Tables S1–S3, Figs. 2 and 3, S1–S7). Our results are also in agreement with the earlier reports (Jensen and Warming 2015; Kar et al. 2017; Li et al. 2020; Mortensen et al. 2020; Wang et al. 2022), demonstrating that longer carbon chains of PFASs favor stronger interactions with TTR than their shorter-chained counterparts through hydrophobic interactions and thereby enhance binding affinity with TTR and eventually more toxic than the short-chain analogs. According to OECD 2020 report, long-chain PFASs are being progressively replaced with short-chain PFASs’ alternatives in US and Europe (Brendel et al. 2018; OECD 2020; Ren et al. 2016). In our study, we confirmed that long-chain PFASs and sulfur-containing PFASs are more toxic and resistant. Moreover, our study raises a new direction with respect to the toxicity of short-chain PFASs, since the toxicity of short-chain PFASs is not thoroughly studied or well described, in particular with regard to long-term effects, and there are examples of exceptions to the general picture (Brendel et al. 2018; Jensen and Warming 2015). We found that out of 15 short-chain PFASs, almost 67% of compounds had the capacity to displace thyroid hormones from TTR protein. Hence, hazards and health risks associated with short-chain PFASs need to be studied in depth (Fig. 5). Regarding interacting TTR interactions with PFASs, our data revealed that K15, A108, A109, L110, and S117 are the key interacting amino acid residues of TTR which were involved in the majority of molecular interactions with PFAS (Tables S1–S3 and Fig. 5). This observation is consistent with that of the previous studies (Kar et al. 2017; Mortensen et al. 2020). Moreover, it is prominent that H-bonds and hydrophobic interactions drive PFASs binding with TTR.

Analysis of binding energy and molecular interactions of TTR variants with PFASs confirmed that the identified amino acid residues, e.g., K15, L110, and S117 are the key residues for PFAS interactions with TTR (Table 1). In this regard, analysis of single amino acid polymorphisms/substitutions in the human population showed that various substitutions, e.g., A108T, A108Y/L110E, A109T, L110M, L110R, and T119M are associated with various diseases such as amyloidogenic transthyretin amyloidosis and peripheral neuropathy (Table S18). It is noteworthy that L110 is one of the key interacting residues with PFASs. This observation would point out the potential structure–function relationship between the TTR variant and toxicity mechanism for PFASs via protein-ligands interaction-mediated biochemical changes, and epigenetic changes, such as DNA methylation (Fenton et al. 2021; Kim et al. 2021; Xu et al. 2022).

One of the most important aspects of toxicology is managing and mitigating environmental pollution and assessing toxicity in other species, along with public health concerns. In this respect, TTR homolog analysis revealed identified key residues in human TTR, e.g., K15, A108, A109, L110, S117, and T119 for interacting with PFASs are conserved among human and other vertebrates, e.g., *Pan troglodytes*, *Macaca fuscata*, *Pongo abelii*, *Rattus norvegicus*, *Danio rerio*, *Xenopus tropicalis*. Moreover, TTR homologs are also observed in invertebrates, e.g., *Caenorhabditis elegans*, *Daphnia pulex*, and *Drosophila navojoa* (flies) (Fig. 4). As conserved protein sequences are known to have identical amino acid residues at analogous domains of the protein structure with similar functions (Konate et al. 2019; Schaefer et al. 2014), the protein function of TTR might be conserved among vertebrates (Pramanik et al. 2017; Sharan et al. 2005). These results strongly support that PFAS-mediated toxicity to human TTR can be used as a reference to predict toxicity to other vertebrates. However, it is crucial to consider the interspecies proteomic analysis across a large sample of species to provide further insights into the PFAS toxicity toward TTR throughout the entire phylogenetic tree of life for the sustainable management of ecosystems.

## Conclusion

In summary, the results of the present study establish a discrete but comprehensive toxicity pattern of long-chain and short-chain PFASs toward TTR. Obtained results disclosed that the binding potency of a PFAS relies on its carbon chain length and functional group, in which longer carbon chains of PFASs and sulfur-containing PFASs favor stronger interactions with TTR than their shorter-chained counterparts. Moreover, these results pointed out the thyroid-disrupting effects of short-chain PFASs and their in-depth toxicity profile needs to be studied to understand the underlying biochemical processes under physiological and pathophysiological conditions. Furthermore, we identified and highlighted the significance of residues K15, A108, L110, S117, and T119 in establishing H-bonding and hydrophobic interaction networks with PFASs. Interestingly, TTR homology analysis strengthens the hypothesis that the thyroid-disrupting effects of PFASs are not only limited to humans, but to other species as well. Taken together, the data presented here facilitate molecular feature-based toxicity prediction of chemicals depending on their binding affinity with biomolecules.

## Supplementary Information

Below is the link to the electronic supplementary material.

Supplementary file1 (DOCX 64353 KB)

Supplementary file2 (DOCX 73 KB)

## Data Availability

All the necessary data is included in the Supplementary material. Any data that are not included will be available on request.

## Abbreviations

AA: Amino acid
CSF: Cerebrospinal fluid
NCBI: National Center for Biotechnology Information
H-bond: Hydrogen bond
OECD: Organisation for Economic Co-operation and Development
PFAS: Per- and polyfluoroalkyl substances
PFOS: Perfluorooctane sulfonate
PFOA: Perfluorooctanoic acid
PPARγ: Peroxisome proliferator-activated receptor gamma
RBP: Retinol-binding protein
TH: Thyroid Hormones
TPO: Thyroid peroxidase
TTR: Transthyretin
T4: Thyroxine
T3: Triiodothyronine
A: Alanine
K: Lysine
L: Leucine
E: Glutamate
T: Threonine
V: Valine
P: Proline
S: Serine
M: Methionine
G: Glycine

## References

[CR1] Aleshire SL, Bradley CA, Richardson LD, Parl FF (1983). Localization of human prealbumin in choroid plexus epithelium. J Histochem Cytochem.

[CR2] Almeida NM, Yi E, Wilson AK (2021). Binding of per-and polyfluoro-alkyl substances to peroxisome proliferator-activated receptor gamma. ACS Omega.

[CR3] Association AT (2019) https://www.thyroid.org/media-main/press-room/. Baillière, Tindall and Cox

[CR4] Bekircan-Kurt CE, Yilmaz E, Arslan D (2022). The functional and structural evaluation of small fibers in asymptomatic carriers of TTR p. Val50Met (Val30Met) mutation. Neuromuscul Disord.

[CR5] Berg V, Nost TH, Hansen S (2015). Assessing the relationship between perfluoroalkyl substances, thyroid hormones and binding proteins in pregnant women; a longitudinal mixed effects approach. Environ Int.

[CR6] Bikadi Z, Hazai E (2009). Application of the PM6 semi-empirical method to modeling proteins enhances docking accuracy of AutoDock. J Cheminform.

[CR7] Blake BE, Pinney SM, Hines EP, Fenton SE, Ferguson KK (2018). Associations between longitudinal serum perfluoroalkyl substance (PFAS) levels and measures of thyroid hormone, kidney function, and body mass index in the Fernald community cohort. Environ Pollut.

[CR8] Brendel S, Fetter E, Staude C, Vierke L, Biegel-Engler A (2018). Short-chain perfluoroalkyl acids: environmental concerns and a regulatory strategy under REACH. Environ Sci Eur.

[CR10] Brent GA (2012). Mechanisms of thyroid hormone action. J Clin Investig.

[CR11] Brown-Leung JM, Cannon JR (2022). Neurotransmission targets of per-and polyfluoroalkyl substance neurotoxicity: mechanisms and potential implications for adverse neurological outcomes. Chem Res Toxicol.

[CR12] Buck RC, Franklin J, Berger U (2011). Perfluoroalkyl and polyfluoroalkyl substances in the environment: terminology, classification, and origins. Integr Environ Assess Manag.

[CR13] Calafat AM, Kato K, Hubbard K, Jia T, Botelho JC, Wong LY (2019). Legacy and alternative per- and polyfluoroalkyl substances in the U.S. general population: paired serum-urine data from the 2013–2014 national health and nutrition examination survey. Environ Int..

[CR14] Camdzic M, Aga DS, Atilla-Gokcumen GE (2022). Cellular interactions and fatty acid transporter CD36-mediated uptake of per-and polyfluorinated alkyl substances (PFAS). Chem Res Toxicol.

[CR15] Schrenk D, Bignami M, Chain EPoCitF (2020). Risk to human health related to the presence of perfluoroalkyl substances in food. EFSA J.

[CR16] Chambers WS, Hopkins JG, Richards SM (2021). A review of per- and polyfluorinated alkyl substance impairment of reproduction. Front Toxicol.

[CR17] Coniglio AC, Segar MW, Loungani RS (2022). Transthyretin V142I genetic variant and cardiac remodeling, injury, and heart failure risk in black adults. JACC Heart Fail.

[CR18] Coperchini F, Croce L, Ricci G (2021). Thyroid disrupting effects of old and new generation PFAS. Front Endocrinol.

[CR19] Cousins DK, Kong D, Vestergren R (2011). Reconciling measurement and modelling studies of the sources and fate of perfluorinated carboxylates. Environ Chem.

[CR20] Cui DN, Li XR, Quinete N (2020). Occurrence, fate, sources and toxicity of PFAS: what we know so far in Florida and major gaps. Trac-Trend Anal Chem.

[CR21] Dallaire R, Dewailly E, Pereg D, Dery S, Ayotte P (2009). Thyroid function and plasma concentrations of polyhalogenated compounds in Inuit adults. Environ Health Perspect.

[CR500] Fenton SE, Ducatman A, Boobis A (2021). Per- and polyfluoroalkyl substance toxicity and human health review: current state of knowledge and strategies for informing future research. Environ Toxicol Chem.

[CR24] Frumkin H (2001). Beyond toxicity: human health and the natural environment. Am J Prev Med.

[CR25] Godlewska M, Banga PJ (2019). Thyroid peroxidase as a dual active site enzyme: focus on biosynthesis, hormonogenesis and thyroid disorders of autoimmunity and cancer. Biochimie.

[CR26] Han W, Gao Y, Yao Q (2018). Perfluoroalkyl and polyfluoroalkyl substances in matched parental and cord serum in Shandong, China. Environ Int.

[CR27] Hennebry SC, Wright HM, Likic VA, Richardson SJ (2006). Structural and functional evolution of transthyretin and transthyretin-like proteins. Proteins.

[CR28] Hollingsworth SA, Dror RO (2018). Molecular dynamics simulation for all. Neuron.

[CR29] Hospital A, Goni JR, Orozco M, Gelpi JL (2015). Molecular dynamics simulations: advances and applications. Adv Appl Bioinform Chem.

[CR30] Huey R, Morris GM (2008). Using AutoDock 4 with AutoDocktools: a tutorial. Scripps Res Inst USA.

[CR31] Ikegami K, Refetoff S, Van Cauter E, Yoshimura T (2019). Interconnection between circadian clocks and thyroid function. Nat Rev Endocrinol.

[CR32] Janson G, Paiardini A (2021). PyMod 3: a complete suite for structural bioinformatics in PyMOL. Bioinformatics.

[CR33] Jensen AA, Warming M (2015). Short-chain polyfluoroalkyl substances (PFAS). DEPA.

[CR34] Kar S, Sepulveda MS, Roy K, Leszczynski J (2017). Endocrine-disrupting activity of per- and polyfluoroalkyl substances: exploring combined approaches of ligand and structure based modeling. Chemosphere.

[CR35] Kato K, Wong LY, Jia LT, Kuklenyik Z, Calafat AM (2011). Trends in exposure to polyfluoroalkyl chemicals in the U.S. Population: 1999–2008. Environ Sci Technol.

[CR36] Kim KS, Friesner RA (1997). Hydrogen bonding between amino acid backbone and side chain analogues: a high-level ab initio study. J Am Chem Soc.

[CR37] Kim HJ, Koedrith P, Seo YR (2015). Ecotoxicogenomic approaches for understanding molecular mechanisms of environmental chemical toxicity using aquatic invertebrate, *Daphnia* model organism. Int J Mol Sci.

[CR38] Kim S, Thiessen PA, Bolton EE (2016). PubChem substance and compound databases. Nucleic Acids Res.

[CR39] Kim S, Chen J, Cheng TJ (2019). PubChem 2019 update: improved access to chemical data. Nucleic Acids Res.

[CR40] Kim S, Thapar I, Brooks BW (2021). Epigenetic changes by per-and polyfluoroalkyl substances (PFAS). Environ Pollut.

[CR41] Klecha AJ, Genaro AM, Lysionek AE, Caro RA, Coluccia AG, Cremaschi GA (2000). Experimental evidence pointing to the bidirectional interaction between the immune system and the thyroid axis. Int J Immunopharmacol.

[CR42] Kockel L, Homsy JG, Bohmann D (2001). Drosophila AP-1: lessons from an invertebrate. Oncogene.

[CR43] Konate MM, Plata G, Park J, Usmanova DR, Wang H, Vitkup D (2019). Molecular function limits divergent protein evolution on planetary timescales. Elife.

[CR44] Krieger E, Vriend G (2014). YASARA view-molecular graphics for all devices-from smartphones to workstations. Bioinformatics.

[CR45] Kwiatkowski CF, Andrews DQ, Birnbaum LS (2020). Scientific basis for managing PFAS as a chemical class. Environ Sci Technol Lett.

[CR46] Ladunga I (2009). Finding homologs in amino acid sequences using network BLAST searches. Curr Protoc Bioinform.

[CR47] Land H, Humble MS (2018). YASARA: A tool to obtain structural guidance in biocatalytic investigations. Methods Mol Biol.

[CR48] Laskowski RA, Swindells MB (2011). LigPlot+: multiple ligand-protein interaction diagrams for drug discovery. J Chem Inf Model.

[CR49] Lau C, Anitole K, Hodes C, Lai D, Pfahles-Hutchens A, Seed J (2007). Perfluoroalkyl acids: a review of monitoring and toxicological findings. Toxicol Sci.

[CR50] Lehman-McKeeman LD, Armstrong LE (2022). Biochemical and molecular basis of toxicity Haschek and Rousseaux’s handbook of toxicologic pathology.

[CR51] Li F, Duan J, Tian S (2020). Short-chain per-and polyfluoroalkyl substances in aquatic systems: occurrence, impacts and treatment. Chem Eng J.

[CR52] Lindstrom AB, Strynar MJ, Libelo EL (2011). Polyfluorinated compounds: past, present, and future. Environ Sci Technol.

[CR53] Lipton J, Kleemann G, Ghosh R, Lints R, Emmons SW (2004). Mate searching in * Caenorhabditis * elegans: a genetic model for sex drive in a simple invertebrate. J Neurosci.

[CR54] Liu Y, Wang J, Fang X, Zhang H, Dai J (2011). The thyroid-disrupting effects of long-term perfluorononanoate exposure on zebrafish (*Danio*
*rerio*). Ecotoxicology.

[CR55] Liz MA, Coelho T, Bellotti V, Fernandez-Arias MI, Mallaina P, Obici L (2020). A narrative review of the role of transthyretin in health and disease. Neurol Ther.

[CR56] Lopez-Espinosa MJ, Mondal D, Armstrong B, Bloom MS, Fletcher T (2012). Thyroid function and perfluoroalkyl acids in children living near a chemical plant. Environ Health Perspect.

[CR57] Martin JW, Mabury SA, O'Brien PJ (2005). Metabolic products and pathways of fluorotelomer alcohols in isolated rat hepatocytes. Chem Biol Interact.

[CR58] Melzer D, Rice N, Depledge MH, Henley WE, Galloway TS (2010). Association between serum perfluorooctanoic acid (PFOA) and thyroid disease in the U.S. National health and nutrition examination survey. Environ Health Perspect.

[CR59] Mortensen Å-K, Mæhre S, Kristiansen K (2020). Homology modeling to screen for potential binding of contaminants to thyroid hormone receptor and transthyretin in glaucous gull (*Larus*
*hyperboreus*) and herring gull (*Larus*
*argentatus*). Comput Toxicol.

[CR60] Mounika B, Brahmaiah B, Ramesh M, Bhavaneshwari K, Anantha Lakshmi T, Nama S (2013). Review on thyroid disorders. Int J Pharm Res Bio-Sci.

[CR61] Mughal BB, Fini JB, Demeneix BA (2018). Thyroid-disrupting chemicals and brain development: an update. Endocr Connect.

[CR62] Mullur R, Liu YY, Brent GA (2014). Thyroid hormone regulation of metabolism. Physiol Rev.

[CR63] Neagu M, Constantin C, Bardi G, Duraes L (2021). Adverse outcome pathway in immunotoxicity of perfluoroalkyls. Curr Opin Toxicol.

[CR64] O'Boyle NM, Banck M, James CA, Morley C, Vandermeersch T, Hutchison GR (2011). Open babel: an open chemical toolbox. J Cheminform.

[CR65] OECD (2020) PFASs and Alternatives in Food Packaging (Paper and Paperboard) Report on the Commercial Availability and Current Uses, OECD Series on Risk Management. EHS OECD No. 58.

[CR66] Olsen GW, Mair DC, Lange CC (2017). Per- and polyfluoroalkyl substances (PFAS) in American red cross adult blood donors, 2000–2015. Environ Res.

[CR67] Parcha V, Malla G, Ivin MR (2022). Association of transthyretin Val122Ile variant with incident heart failure among black individuals. JAMA.

[CR68] Power D, Elias N, Richardson S, Mendes J, Soares C, Santos C (2000). Evolution of the thyroid hormone-binding protein, transthyretin. Gen Comp Endocrinol.

[CR69] Pramanik S, Kutzner A, Heese K (2017). Livebearing or egg-laying mammals: 27 decisive nucleotides of FAM168. Biosci Trends.

[CR70] Prapunpoj P, Leelawatwattana L (2009). Evolutionary changes to transthyretin: structure-function relationships. FEBS J.

[CR71] Prento P (2007). The role of glycine and prolines in connective tissue fiber staining with hydrogen bonding dyes. Biotech Histochem.

[CR72] Procter JB, Carstairs GM, Soares B (2021). Alignment of biological sequences with jalview. Methods Mol Biol.

[CR73] Rabah SA, Gowan IL, Pagnin M, Osman N, Richardson SJ (2019). Thyroid hormone distributor proteins during development in vertebrates. Front Endocrinol.

[CR74] Raghu P, Sivakumar B (2004). Interactions amongst plasma retinol-binding protein, transthyretin and their ligands: implications in vitamin a homeostasis and transthyretin amyloidosis. Biochim Biophys Acta Proteins Proteom.

[CR75] Ren X-M, Qin W-P, Cao L-Y (2016). Binding interactions of perfluoroalkyl substances with thyroid hormone transport proteins and potential toxicological implications. Toxicology.

[CR77] Rericha Y, Cao D, Truong L, Simonich M, Field JA, Tanguay RL (2021). Behavior effects of structurally diverse per-and polyfluoroalkyl substances in zebrafish. Chem Res Toxicol.

[CR78] Richardson SJ, Wijayagunaratne RC, D'Souza DG, Darras VM, Van Herck SL (2015). Transport of thyroid hormones via the choroid plexus into the brain: the roles of transthyretin and thyroid hormone transmembrane transporters. Front Neurosci.

[CR79] Rickard BP, Rizvi I, Fenton SE (2022). Per- and poly-fluoroalkyl substances (PFAS) and female reproductive outcomes: PFAS elimination, endocrine-mediated effects, and disease. Toxicology.

[CR80] Schaefer MH, Yang J-S, Serrano L, Kiel C (2014). Protein conservation and variation suggest mechanisms of cell type-specific modulation of signaling pathways. PLOS Comput Biol.

[CR81] Seeliger D, de Groot BL (2010). Ligand docking and binding site analysis with PyMOL and Autodock/Vina. J Comput Aided Mol Des.

[CR82] Sharan R, Suthram S, Kelley RM (2005). Conserved patterns of protein interaction in multiple species. Proc Natl Acad Sci USA.

[CR83] Sievers F, Higgins DG (2014). Clustal omega. Curr Protoc Bioinform.

[CR84] Takahashi Y, Ohashi N, Takasone K (2022). CSF/plasma levels, transthyretin stabilisation and safety of multiple doses of tolcapone in subjects with hereditary ATTR amyloidosis. Amyloid.

[CR85] Thibodeaux JR, Hanson RG, Rogers JM (2003). Exposure to perfluorooctane sulfonate during pregnancy in rat and mouse. I: maternal and prenatal evaluations. Toxicol Sci.

[CR86] Tomar D, Khan T, Singh RR (2012). Crystallographic study of novel transthyretin ligands exhibiting negative-cooperativity between two thyroxine binding sites. Plos One.

[CR87] Umesh KD, Selvaraj C, Singh SK, Dubey VK (2021). Identification of new anti-nCoV drug chemical compounds from Indian spices exploiting SARS-CoV-2 main protease as target. J Biomol Struct Dyn.

[CR88] Vanderlinde T, Dupim EG, Nazario-Yepiz NO, Carvalho AB (2019). An improved genome assembly for * Drosophila * navojoa, the basal species in the mojavensis cluster. J Hered.

[CR89] Vieira M, Saraiva MJ (2014). Transthyretin: a multifaceted protein. Biomol Concepts.

[CR90] Wang Z, Cousins IT, Scheringer M, Buck RC, Hungerbuhler K (2014). Global emission inventories for C4–C14 perfluoroalkyl carboxylic acid (PFCA) homologues from 1951 to 2030, part II: the remaining pieces of the puzzle. Environ Int.

[CR91] Wang Y, Kim J, Huang C-H (2022). Occurrence of per-and polyfluoroalkyl substances in water: a review. Environ Sci Water Res Technol.

[CR92] Webster GM, Venners SA, Mattman A, Martin JW (2014). Associations between perfluoroalkyl acids (PFASs) and maternal thyroid hormones in early pregnancy: a population-based cohort study. Environ Res.

[CR93] Weiss JM, Andersson PL, Lamoree MH, Leonards PE, van Leeuwen SP, Hamers T (2009). Competitive binding of poly- and perfluorinated compounds to the thyroid hormone transport protein transthyretin. Toxicol Sci.

[CR94] Williams GR (2008). Neurodevelopmental and neurophysiological actions of thyroid hormone. J Neuroendocrinol.

[CR95] Wojtczak A, Neumann P, Cody V (2001). Structure of a new polymorphic monoclinic form of human transthyretin at 3 A resolution reveals a mixed complex between unliganded and T4-bound tetramers of TTR. Acta Crystallogr D Biol Crystallogr.

[CR96] Xu Y, Lindh CH, Fletcher T, Jakobsson K, Engström K (2022). Perfluoroalkyl substances influence DNA methylation in school-age children highly exposed through drinking water contaminated from firefighting foam: a cohort study in Ronneby, Sweden. Environ Epigenet.

[CR97] Yee AW, Aldeghi M, Blakeley MP (2019). A molecular mechanism for transthyretin amyloidogenesis. Nat Commun.

[CR98] Yu WG, Liu W, Jin YH (2009). Effects of perfluorooctane sulfonate on rat thyroid hormone biosynthesis and metabolism. Environ Toxicol Chem.

[CR99] Zeng Z, Song B, Xiao R (2019). Assessing the human health risks of perfluorooctane sulfonate by in vivo and in vitro studies. Environ Int.

