# Supplementary material for: In silico analysis decodes transthyretin (TTR) binding and thyroid disrupting effects of per- and polyfluoroalkyl substances (PFAS)
Source: Arch Toxicol. 2022 Dec 25;97(3):755–68. doi: 10.1007/s00204-022-03434-8 (PMC9968702; doi:10.1007/s00204-022-03434-8)
Supplement: Supplementary file 1 — Supplementary file1 (DOCX 64353 KB) [file 204_2022_3434_MOESM1_ESM.docx]

***In silico* analysis decodes Transthyretin (TTR) binding and Thyroid Disrupting Effects of Per- and polyfluoroalkyl substances (PFAS)**

Rupal Dharpure^1^, Subrata Pramanik^1, *^, Ajay Pradhan^2, *^

^1^ Jyoti and Bhupat Mehta School of Health Science and Technology, Indian Institute of Technology Guwahati, Guwahati, Assam 781039, India

^2^ Biology, The Life Science Center, School of Science and Technology, Örebro University, SE-70182 Örebro, Sweden

*Corresponding authors: Ajay Pradhan ([ajay.pradhan@oru.se](mailto:ajay.pradhan@oru.se)), Subrata Pramanik ([subrata.pramanik@iitg.ac.in](mailto:subrata.pramanik@iitg.ac.in), [subrata.biocell@gmail.com](mailto:subrata.biocell@gmail.com))

**Figure S1. Molecular interactions of long-chain PFASs with TTR.** H-bonds observed between TTR and PFASs are indicated by green dashed lines along with bond length, while hydrophobic interactions are represented by brown arcs with spokes radiating toward PFASs. Molecular interactions of **(A)** Perfluorotributyl amine (-9.2 kcal/mol), **(B)** Perfluorodecane sulfonic acid (-9.1 kcal/mol), and **(C)** Perfluorononane sulfonic acid (-8.7 kcal/mol) with TTR are shown, respectively.

**Figure S2. Molecular interactions of long-chain PFASs with TTR.** H-bonds observed between protein and ligand is indicated by green dashed lines along with bond length, while hydrophobic interactions are represented by a brown arc with spokes radiating towards ligand. Molecular interactions of (**A**) Perfluorodecanoic acid (-8.7 kcal/mol), (**B**) Perfluorononanoic acid (-8.5 kcal/mol), and (**C**) Perfluorooctanesulfonic acid (-8.2 kcal/mol) with TTR are shown, respectively.

**Figure S3. Molecular interactions of long-chain PFASs with TTR.** H-bonds observed between protein and ligand is indicated by green dashed lines along with bond length, while hydrophobic interactions are represented by a brown arc with spokes radiating towards ligand. Molecular interactions of (**A**) Perfluorooctanoic acid (-8 kcal/mol) and (**B**) Perfluorohenptanoic acid (-7.6 kcal/mol) with TTR are shown, respectively.

**Figure S4. Molecular interactions of long-chain PFASs with TTR.** H-bonds observed between protein and ligand is indicated by green dashed lines along with bond length, while hydrophobic interactions are represented by a brown arc with spokes radiating towards ligand. Molecular interactions of (**A**) N-Methylperfluorooctanesulfonamidoethanol (-7.6 kcal/mol) and (**B**) 6:2 Fluorotelomer alcohol (-7.5 kcal/mol) with TTR are shown, respectively.

**Figure S5. Molecular interactions of short-chain PFASs with TTR.** H-bonds observed between protein and ligand is indicated by green dashed lines along with bond length, while hydrophobic interactions are represented by a brown arc with spokes radiating towards ligand. Molecular interactions of (**A**) Perfluorohexane sulfonic acid (-7.2 kcal/mol), (**B**) Perfluorobutane sulfonic acid (-6.9 kcal/mol), and (**C**) Perfluoropentanoic acid (-6.8 kcal/mol) with TTR are shown, respectively.

**Figure S6. Molecular interactions of short-chain PFASs with TTR.** H-bonds observed between protein and ligand is indicated by green dashed lines along with bond length, while hydrophobic interactions are represented by a brown arc with spokes radiating towards ligand. Molecular interactions of ((**A**) Perfluoropentane sulfonic acid (-6.8 kcal/mol), (**B**) 4:2 Fluorotelomer alcohol (-6.8 kcal/mol), and (**C**) Perfluorobutyl phosphonate (-6.7 kcal/mol) with TTR are shown, respectively.

**Figure S7. Molecular interactions of short-chain PFASs with TTR.** H-bonds observed between protein and ligand is indicated by green dashed lines along with bond length, while hydrophobic interactions are represented by a brown arc with spokes radiating towards ligand. Molecular interactions of (**A**) Perfluorobutyl iodide (-6.3 kcal/mol), (**B**) Perfluorobutanoic acid (-6.0 kcal/mol), and (**C**) Perfluoropropane sulfonic acid (-5.9 kcal/mol) with TTR are shown, respectively.

**Figure S8.** **3D Molecular interactions of long-chain PFASs with TTR.** H-bonds observed between PFAS and amino acid residue from TTR is indicated by orange, teal solid lines along with bond length, while hydrophobic interactions are represented by grey spheres. Amino acid residues around the PFAS (**A**) Perfluorotetradecanoic acid (-9.8 kcal/mol), (**B**) Perfluorododecanoic acid (-9.4 kcal/mol), and (**C**) Perfluoroundecanoic acid (-9.3 kcal/mol) with TTR are shown, respectively.

**Figure S9. 3D Molecular interactions of long-chain PFASs with TTR.** H-bonds observed between PFAS and amino acid residue from TTR is indicated by orange, teal solid lines along with bond length, while hydrophobic interactions are represented by grey spheres. Amino acid residues around the PFAS (**A**) 2H-perfluoro-2-octenoic acid (-7.4 kcal/mol), (**B**) 7H-Perfluoroheptanoic acid (-7.4 kcal/mol), and (**C**) 5:2 fluorotelomer alcohol (-6.7 kcal/mol) with TTR are shown, respectively.

**Figure S10. 3D Molecular interactions of short-chain PFASs with TTR.** H-bonds observed between PFAS and amino acid residue from TTR is indicated by orange, teal solid lines along with bond length, while hydrophobic interactions are represented by grey spheres. Amino acid residues around the PFAS (**A**) Perfluorohexanoic acid (-7.4 kcal/mol), (**B**) Perfluorohexyl phosphonate (-7.3 kcal/mol), and (**C**) Perfluorohexane sulfonate (-7.3 kcal/mol) with TTR are shown, respectively.

**Figure S11. 3D Molecular interactions of short-chain PFASs with TTR.** H-bonds observed between PFAS and amino acid residue from TTR is indicated by orange, teal solid lines along with bond length, while hydrophobic interactions are represented by grey spheres. Amino acid residues around the PFAS (**A**) Perfluoroethane sulfonic acid (-4.9 kcal/mol), (**B**) Trifluoromethane sulfonic acid (-4.2 kcal/mol), and (**C**) Trifluoroacetic acid (-4.0 kcal/mol) with TTR are shown, respectively.


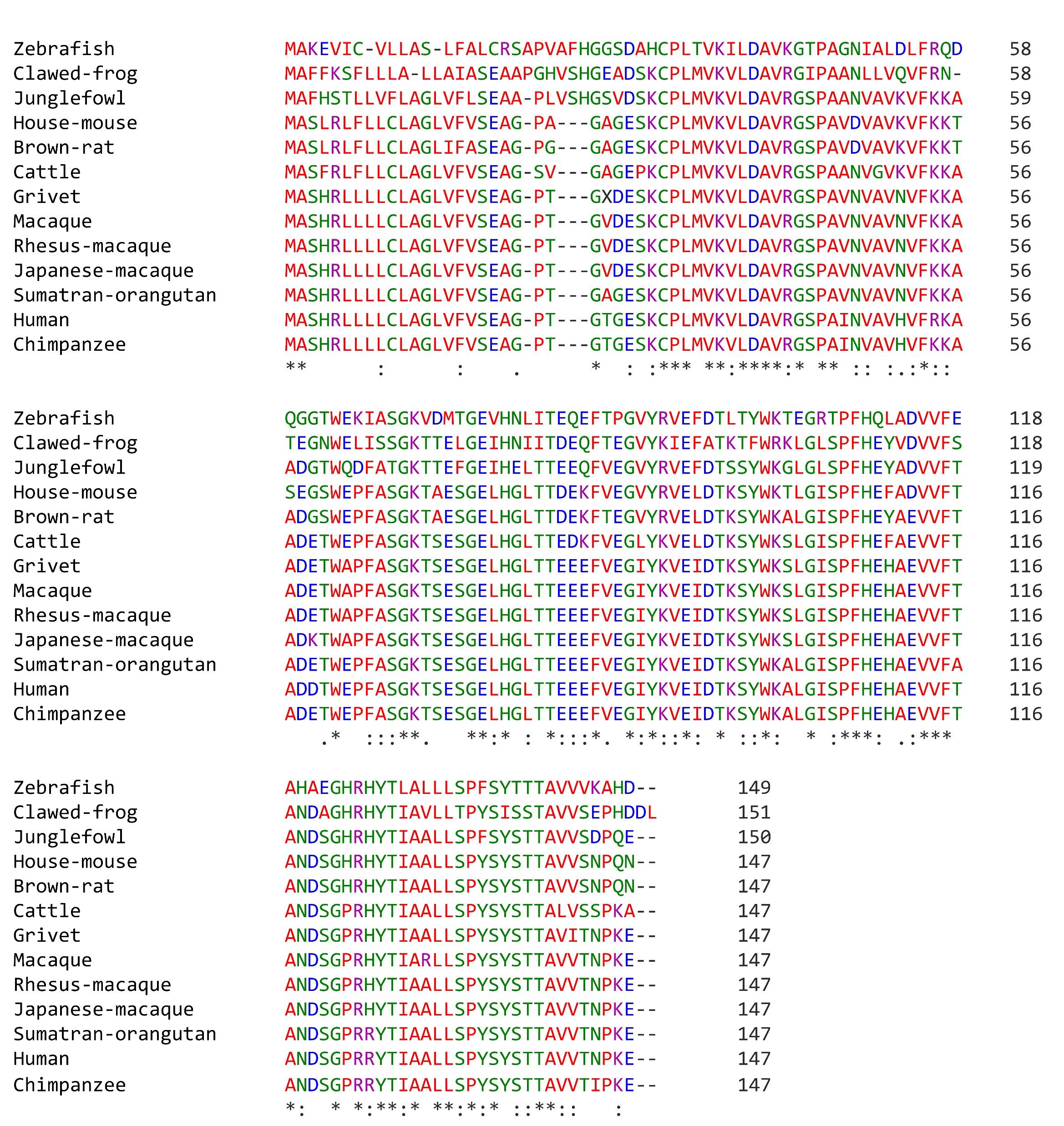


**Figure S12**. **TTR homologs analysis**. Comparison of amino acid sequence of TTR homologs among 13 different vertebrates are shown.
