# Supplementary material for: In silico analysis decodes transthyretin (TTR) binding and thyroid disrupting effects of per- and polyfluoroalkyl substances (PFAS)
Source: Arch Toxicol. 2022 Dec 25;97(3):755–68. doi: 10.1007/s00204-022-03434-8 (PMC9968702; doi:10.1007/s00204-022-03434-8)
Supplement: Supplementary file 2 — Supplementary file2 (DOCX 73 KB) [file 204_2022_3434_MOESM2_ESM.docx]

**Supplementary Table**

***In silico* analysis decodes Transthyretin (TTR) binding and Thyroid Disrupting Effects of Per- and polyfluoroalkyl substances (PFAS)**

Rupal Dharpure^1^, Subrata Pramanik^1, *^, Ajay Pradhan^2, *^

^1^Jyoti and Bhupat Mehta School of Health Science and Technology, Indian Institute of Technology Guwahati, Guwahati, Assam 781039, India

^2^ Biology, The Life Science Center, School of Science and Technology, Örebro University, SE-70182 Örebro, Sweden

*Corresponding authors: Ajay Pradhan ([ajay.pradhan@oru.se](mailto:ajay.pradhan@oru.se)), Subrata Pramanik ([subrata.pramanik@iitg.ac.in](mailto:subrata.pramanik@iitg.ac.in), [subrata.biocell@gmail.com](mailto:subrata.biocell@gmail.com))

**Table S1.** Binding energy, interacting amino acids, and type of interactions of natural ligands with TTR

| **Natural ligands** | **Binding Energy (kcal/mol)** | **Interacting amino acids** | **Type of interactions with Chain A and Chain C** |
| --- | --- | --- | --- |
| Thyroxine (T4) | -6.2 | **Chain A**: K15; **Chain C**: K15, L17, E54, A108, A109, L110 | **H-bonds:** H-bond1: L110(C), H-bond2: A109(C); **Hydrophobic**:  K15(A), K15(C), L17(C), E54(C), A108(C), A109(C), L110(C) |
| Triiodothyronin (T3) | -6.2 | **Chain A**: K15, L17, T106, A108, V121, T123; **Chain C**: K15, P24, S52, A108 | **H-bonds:** H-bond1: T106(A), H-bond2: S52(C), H-bond3: T123(A); **Hydrophobic**: K15(A), K15(C), L17(A), T106(A), A108(A), V121(A), T123(A), P24(C), S52(C), A108(C) |

**Table S2.** Binding energy, interacting amino acids, and type of interactions of long-chain PFAS with TTR

| **Sl. No.** | **Long-chain PFAS** | **Binding Energy (kcal/mol)** | **Interacting amino acids** | **Type of interactions with Chain A and Chain C** |
| --- | --- | --- | --- | --- |
| 1 | Perfluorotetradecanoic acid | -9.8 | **Chain A**: K15, T106, A108, A109, L110, S117; **Chain C**: K15 | **H-bonds:** H-bond1: S117(A), H-bond2: S117(A), H-bond3: L110(A), H-bond4: T106(A), H-bond5: K15(C), H-bond6: K15(C), H-bond7: K15(C); **Hydrophobic**: K15(A), A108(A), A109(A), L110(A), S117(A), T118(A), T119(A) |
| 2 | Perfluorododecanoic acid | -9.4 | **Chain A**: K15, A108, T119; **Chain C**: T106, A108, L110, S117, T119 | **H-bonds:** H-bond1: T106(C), H-bond2: T106(C), H-bond3: K15(A), H-bond4: L110(C), H-bond5: K15(A), H-bond6: S117(C);  **Hydrophobic**: K15(A), A108(A), T119(A), T106(C), A108(C), L110(C), T119(C) |
| 3 | Perfluoroundecanoic acid | -9.3 | **Chain A**: K15, T106, A108, A109, L110, S117, T118, T119, V121;  **Chain C**: K15, L110. | **H-bonds:** H-bond1: K15(C), H-bond2: L110(A), H-bond3: S117(A), H-bond4: A108(A), H-bond5: S117(A); **Hydrophobic**: K15(A), T106(A), A108(A), A109(A), L110(A), S117(A), T118(A), T119(A), L110(C) |
| 4 | Perfluorotributyl amine (PTBA) | -9.2 | **Chain A**: K15, L17, A108; **Chain C**: L17, A108, L110, S117, V121 | **H-bonds:** H-bond1: L110(C), H-bond2: S117(C); **Hydrophobic**:  K15(A), L17(A), A108(A), L17(C), A108(C), V121(C) |
| 5 | Perfluorodecane sulfonic acid (PFDS) | -9.1 | **Chain A**: K15, A108, T119;  **Chain C**: T106, A108, S117 | **H-bonds:** H-bond1: K15(A), H-bond2: K15(A), H-bond3: S117(C), H-bond4: K15(A); **Hydrophobic**: K15(A), A108(A), T119(A), T106(C), A108(C) |
| 6 | Perfluorononane sulfonic acid (PFNS) | -8.7 | **Chain A**: L17, T106, V121; **Chain C**: K15, L17, L110, S117, T119 | **H-bonds:** H-bond1: K15(C), H-bond2: S117(C), H-bond3: T119(C); **Hydrophobic**: L17(A), T106(A), V121(A), K15(C), L17(C), L110(C), S117(C), T119(C) |
| 7 | Perfluorodecanoic acid | -8.7 | **Chain A**: K15, L17, A108, T119; **Chain C**: T106, A108, L110, S117, T119 | **H-bonds:** H-bond1: L110(C), H-bond2: S117(C); **Hydrophobic**:  K15(A), L17(A), A108(A), T119(A), T106(C), A108(C), L110(C), S117(C), T119(C) |
| 8 | Perfluorononanoic acid (PFNA) | -8.5 | **Chain A**: K15, A109, L110, S117, T118, T119; **Chain C**: L17, A108, L110 | **H-bonds:** H-bond1: L110(A), H-bond2: S117(A), H-bond3: S117(A), H-bond4: T119(A), H-bond5: S117(A); **Hydrophobic**:  K15(A), A109(A), L110(A), S117(A), T118(A), T119(A),  L17(C), A108(C), L110(C) |
| 9 | Perfluorooctanesulfonic acid (PFOS) | -8.2 | **Chain A**: K15, L110, S117; **Chain C**: A108, L110, S117, T119 | **H-bonds:** H-bond1: S117(C), H-bond2: S117(A), H-bond3: S117(C); **Hydrophobic**: K15(A), L110(A), S117(A), A108(C), L110(C), S117(C), T119(C) |
| 10 | Perfluorooctanoic acid (PFOA) | -8 | **Chain A**: K15, A108, A109, L110, S117, T119; **Chain C**: L110 | **H-bonds:** H-bond1: L110(A), H-bond2: S117(A), H-bond3: S117(A); **Hydrophobic**: K15(A), A108(A), A109(A), L110(A), S117(A), T119(A), L110(C) |
| 11 | Perfluorohenptanoic acid (PFHpA) | -7.6 | **Chain A**: K15, L17, A109, L110, S117, T118, T119; **Chain C**: L110, T119 | **H-bonds:** H-bond1: L110(A), H-bond2: S117(A), H-bond3: S117(A), H-bond4: T119(C); **Hydrophobic**: K15(A), L17(A), A109(A), L110(A), S117, T118(A), T119(A), L110(C) |
| 12 | N-Methylperfluorooctanesulfonamidoethanol | -7.6 | **Chain A**: M13, K15, L17, E54, T106, V121; **Chain C**: K15, L17, T119 | **H-bonds:** H-bond1: E54(A), H-bond2: T119(C), H-bond3: K15(C); **Hydrophobic**: M13(A), K15(A), L17(A), E54(A), T106(A), V121(A), K15(C), L17(C) |
| 13 | 6:2 Fluorotelomer alcohol (FTOH) | -7.5 | **Chain A**: L17, A108, A109, L110, S117 T118, T119; **Chain C**: A108, L110, S117, T119. | **H-bonds:** H-bond1: T119(A), H-bond2: S117(A), H-bond3: A108(A), H-bond4: S117(C), H-bond5: T119(C); **Hydrophobic**:  L17(A), A108(A), A109(A), L110(A), S117(A), T118(A), T119(A), A108(C), L110(C), S117(C), T119(C) |
| 14 | 2H-Perfluoro-2-octenoic acid | -7.4 | **Chain A**: K15, L17, A108, A109, S117, T118, T119; **Chain C**: A108, L110 | **H-bonds:** H-bond1: T119(A), H-bond2: S117(A), H-bond3: A108(A), H-bond4: S117(A); **Hydrophobic**: K15(A), L17(A), A108(A), A109(A), S117(A), T118(A), T119(A), A108(C), L110(C) |
| 15 | 7H-Perfluoroheptanoic acid | -7.4 | **Chain A**: K15, L17, A108, A109, L110, S117, T118, T119; **Chain C**: L110, T119 | **H-bonds:** H-bond1: L110(A), H-bond2: S117(A), H-bond3: S117(A), H-bond4: T119(C); **Hydrophobic**: K15(A), L17(A), A108(A), A109(A), L110(A), S117(A), T118(A), T119(A), L110(C) |
| 16 | 5:2 fluorotelomer alcohol | -6.7 | **Chain A**: L17, A108; **Chain C**: L17, A108, A109, S117, T118, T119 | **H-bonds:** H-bond1: T119(C), H-bond2: A109(C), H-bond3: S117(C), H-bond4: A108(C); **Hydrophobic**: L17(A), A108(A), L17(C), A108(C), A109(C), S117(C), T118(C), T119(C) |

**Table S3.** Binding energy, interacting amino acids, and type of interactions of short-chain PFAS with TTR

| **Sl. No.** | **Short-chain PFAS** | **Binding Energy (kcal/mol)** | **Interacting amino acids** | **Type of interactions with Chain A and Chain C** |
| --- | --- | --- | --- | --- |
| 1 | Perfluorohexanoic acid (PFHxA) | -7.4 | **Chain A**: A108, A109, L110, S117, T118, T119; **Chain C**: L17, A108, A109, L110 | **H-bonds:** H-bond1: L110(A), H-bond2: S117(A), H-bond3: A108(A), H-bond4: S117(C), H-bond5: T119(A), H-bond6: S117(A), H-bond7: A109(C), H-bond8: A109(C); **Hydrophobic:**  A108(A), A109(A), L110(A), S117(A), T118(A), T119(A), L17(C), A108(C), A109(C), L110(C) |
| 2 | Perfluorohexyl phosphonate (PFHxPA) | -7.3 | **Chain A**: A109, L110, S117, T118, T119; **Chain C**: A108, L110, T119 | **H-bonds:** H-bond1: L110(A), H-bond2: S117(A), H-bond3: S117(A), H-bond4: S117(A), H-bond5: T119(C); **Hydrophobic**:  A109(A), L110(A), S117(A), T118(A), T119(A), A108(C), L110(C), T119(C) |
| 3 | Perfluorohexane sulfonate | -7.3 | **Chain A**: L17, L110, S117; **Chain C**: L110, S117, T119 | **H-bonds:** H-bond1: S117(A), H-bond2: S117(C); **Hydrophobic**:  L17(A), L110(A), S117(A), L110(C), S117(C), T119(C) |
| 4 | Perfluorohexane sulfonic acid (PFHxS) | -7.2 | **Chain A**: L17, L110, S117; **Chain C**: L110, S117, T119 | **H-bonds:** H-bond1: S117(C), H-bond2: S117(A); **Hydrophobic**:  L17(A), L110(A), S117(A), L110(C), S117(C), T119(C) |
| 5 | Perfluorobutane sulfonic acid (PFBS) | -6.9 | **Chain A**: L17, A108, A109, L110; **Chain C**: A108, A109, L110, S117, T118, T119 | **H-bonds:** H-bond1: S117(C), H-bond2: L110(C), H-bond3: S117(C), H-bond4: T119(C), H-bond5: A109(A), H-bond6: T119(C), H-bond7: L110(C), H-bond8: A109(C); **Hydrophobic**:  L17(A), A108(A), A109(A), L110(A), A108(C), A109(C), L110(C), S117(C), T118(C), T119(C) |
| 6 | Perfluoropentanoic acid (PFPeA) | -6.8 | **Chain A**: L17, A108, A109, L110;  **Chain C**: A108, A109, L110, S117, T119 | **H-bonds:** H-bond1: L110(C), H-bond2: A109(C), H-bond3: S117(C), H-bond4: S117(C), H-bond5: T119(C), H-bond6: L110(A), H-bond7: A109(A); **Hydrophobic**: L17(A), A108(A), A109(A), A108(C), A109(C), S117(C), T119(C) |
| 7 | Perfluoropentane sulfonic acid (PFPeS) | -6.8 | **Chain A**: L17, L110, S117; **Chain C**: A108, L110, S117, T119 | **H-bonds:** H-bond1: S117(A), H-bond2: S117(C); **Hydrophobic**:  L17(A), L110(A), S117(A), A108(C), L110(C), S117(C), T119(C) |
| 8 | 4:2 Fluorotelomer alcohol (4:2 FTOH) | -6.8 | **Chain A**: L17, A109, L110;  **Chain C**: A108, A109, L110, S117, T118, T119 | **H-bonds:** H-bond1: T119(C), H-bond2: A109(C), H-bond3: S117(C), H-bond4: A108(C), H-bond5: L110(A), H-bond6: A109(A); **Hydrophobic**: L17(A), A109(A), A108(C), L110(C), S117(C), T118(C), T119(C) |
| 9 | Perfluorobutyl phosphonate (PFBPA) | -6.7 | **Chain A**: L17, A108, A109, L110; **Chain C**: A108, A109, L110, S117, T118, T119 | **H-bonds:** H-bond1: S117(C), H-bond2: L110(C), H-bond3: S117(C), H-bond4: L110(C), H-bond5: A109(C), H-bond6: A109(C), H-bond7: T119(C), H-bond8: T119(C), H-bond9: A109(A); **Hydrophobic**: L17(A), A108(A), A109(A), L110(A), A108(C), A109(C), L110(C), S117(C), T118(C), T119(C) |
| 10 | Perfluorobutyl iodide (PFBI) | -6.3 | **Chain A**: L17, A109, L110; **Chain C**: T119 | **H-bonds:** H-bond1: L119(C), H-bond2: L110(A), H-bond3: A109(A); **Hydrophobic**: L17(A), A109(A) |
| 11 | Perfluorobutanoic acid (PFBA) | -6.0 | **Chain A**: A108, A109, L110, S117, T118, T119 | **H-bonds:** H-bond1: A108(A), H-bond2: A109(A); **Hydrophobic:**  A108(A), A109(A), L110(A), S117(A), T118(A), T119(A) |
| 12 | Perfluoropropane sulfonic acid (PFPrS) | -5.9 | **Chain A**: L17, A108, A109, L110, T119 | **H-bonds:** H-bond1: L110(A); **Hydrophobic**: L17(A), A108(A), A109(A), L110(A), T119(A) |
| 13 | Perfluoroethane sulfonic acid (PFEtS) | -4.9 | **Chain A**: L17, A108; **Chain C**: L17, A108, L110, T119 | **H-bonds:** H-bond1: T119(C); **Hydrophobic**: L17(A), A108(A), L17(C), A108(C), L110(C), T119(C) |
| 14 | Trifluoromethane sulfonic acid (TFMS) | -4.2 | **Chain C**: L17, A108, A109, L110, S117, T119. | **H-bonds:** H-bond1: L110(C), H-bond2: A109(C), H-bond3: S117(C), H-bond4: L110(C), H-bond5: T119(C); **Hydrophobic:**  L17(C), A108(C), A109(C), L110(C), S117(C), T119(C) |
| 15 | Trifluoroacetic acid (TFA) | -4.0 | **Chain A**: A108, A109, L110, S117, T118, T119; **Chain C**: L110. | **H-bonds:** H-bond1: L110(A), H-bond2: S117(A), H-bond3: A108(A), H-bond4: S117(A), H-bond5: T119(A), H-bond6: T118(A); **Hydrophobic**: A108(A), A109(A), L110(A), S117(A), T118(A), T119(A), L110(C) |

**Table S4.** Important amino acids for molecular interactions between long-, and short-chain PFASs and TTR respectively.

| **Sr.no.** | **Chain A** | **Chain C** |
| --- | --- | --- |
| 1 | Lysine 15 | Lysine 15 |
| 2 | Alanine 108 | Alanine 108 |
| 3 | Alanine 109 | Alanine 109 |
| 4 | Leucine 110 | Leucine 110 |
| 5 | Serine 117 | Serine 117 |

**Table S5.** Effect of amino acid substitution on molecular interactions between TTR and long-chain PFAS, perfluorotetradecanoic acid.

| **Perfluorotetradecanoic acid** | **Wild-type TTR** | **K15G** | **L110G** | **K15G/L110G** | **K15G/L110G/**  **S117G** |
| --- | --- | --- | --- | --- | --- |
| **Binding energy** | -9.8 | -8.8 | -8.8 | -8.2 | -7.9 |
| **Number of Hydrogen bonds** | 7 | 3 | 4 | 3 | 3 |
| **Number of hydrophobic interactions** | 8 | 7 | 8 | 2 | 4 |
| **Total interactions** | **15** | **10** | **12** | **5** | **7** |

**Table S6.** Effect of amino acid substitution on molecular interactions between TTR and long-chain PFAS, perfluorododecanoic acid.

| **Perfluorododecanoic acid** | **Wild-type TTR** | **K15G** | **L110G** | **K15G/L110G** | **K15G/L110G/**  **S117G** |
| --- | --- | --- | --- | --- | --- |
| **Binding energy** | -9.4 | -8.1 | -8.1 | -7.8 | -7.4 |
| **Number of Hydrogen bonds** | 6 | 1 | 0 | 1 | 1 |
| **Number of hydrophobic interactions** | 8 | 7 | 4 | 7 | 3 |
| **Total interactions** | **14** | **8** | **4** | **8** | **4** |

| **Perfluoroundecanoic acid** | **Wild-type TTR** | **K15G** | **L110G** | **K15G/L110G** | **K15G/L110G/**  **S117G** |
| --- | --- | --- | --- | --- | --- |
| **Binding energy** | -9.3 | -8.4 | -8.6 | -7.4 | -7.2 |
| **Number of Hydrogen bonds** | 5 | 2 | 2 | 1 | 1 |
| **Number of hydrophobic interactions** | 14 | 6 | 7 | 5 | 5 |
| **Total interactions** | **19** | **8** | **9** | **6** | **6** |

**Table S7.** Effect of amino acid substitution on molecular interactions between TTR and long-chain PFAS, perfluoroundecanoic acid.

**Table S8.** Effect of amino acid substitution on molecular interactions between TTR and long-chain PFAS, 2H-Perfluoro-2-octenoic acid.

| **2H-Perfluoro-2-octenoic acid** | **Wild-type TTR** | **K15G** | **L110G** | **K15G/L110G** | **K15G/L110G/**  **S117G** |
| --- | --- | --- | --- | --- | --- |
| **Binding energy** | -7.4 | -7.1 | -7 | -6.4 | -6.4 |
| **Number of Hydrogen bonds** | 4 | 2 | 3 | 3 | 3 |
| **Number of hydrophobic interactions** | 11 | 5 | 6 | 5 | 3 |
| **Total interactions** | **15** | **7** | **9** | **8** | **6** |

**Table S9.** Effect of amino acid substitution on molecular interactions between TTR and long-chain PFAS, 7H-perfluoroheptanoic acid.

| **7H-Perfluoroheptanoic acid** | **Wild-type TTR** | **K15G** | **L110G** | **K15G/L110G** | **K15G/L110G/**  **S117G** |
| --- | --- | --- | --- | --- | --- |
| **Binding energy** | -7.4 | -6.9 | -6.5 | -6.5 | -6.0 |
| **Number of Hydrogen bonds** | 4 | 3 | 1 | 2 | 1 |
| **Number of hydrophobic interactions** | 14 | 5 | 4 | 8 | 3 |
| **Total interactions** | **18** | **8** | **5** | **10** | **4** |

**Table S10.** Effect of amino acid substitution on molecular interactions between TTR and long-chain PFAS, 5:2 Fluorotelomer alcohol.

| **5:2 Fluorotelomer alcohol** | **Wild-type TTR** | **K15G** | **L110G** | **K15G/L110G** | **K15G/L110G/**  **S117G** |
| --- | --- | --- | --- | --- | --- |
| **Binding energy** | -6.7 | -6.5 | -6.1 | -5.6 | -5.6 |
| **Number of Hydrogen bonds** | 4 | 3 | 4 | 4 | 3 |
| **Number of hydrophobic interactions** | 13 | 4 | 5 | 3 | 3 |
| **Total interactions** | **17** | **7** | **9** | **7** | **6** |

**Table S11.** Effect of amino acid substitution on molecular interactions between TTR and short-chain PFAS, perfluorohexanoic acid.

| **Perfluorohexanoic acid** | **Wild-type TTR** | **K15G** | **L110G** | **K15G/L110G** | **K15G/L110G/**  **S117G** |
| --- | --- | --- | --- | --- | --- |
| **Binding energy** | -7.4 | -7.0 | -6.3 | -6.4 | -6.1 |
| **Number of Hydrogen bonds** | 8 | 2 | 2 | 2 | 3 |
| **Number of hydrophobic interactions** | 15 | 8 | 5 | 8 | 5 |
| **Total interactions** | **23** | **10** | **7** | **10** | **8** |

**Table S12.** Effect of amino acid substitution on molecular interactions between TTR and short-chain PFAS, perfluorohexyl phosphonate.

| **Perfluorohexyl phosphonate** | **Wild-type TTR** | **K15G** | **L110G** | **K15G/L110G** | **K15G/L110G/**  **S117G** |
| --- | --- | --- | --- | --- | --- |
| **Binding energy** | -7.3 | -6.8 | -7.2 | -6.5 | -6.3 |
| **Number of Hydrogen bonds** | 5 | 4 | 6 | 5 | 6 |
| **Number of hydrophobic interactions** | 13 | 5 | 2 | 3 | 3 |
| **Total interactions** | **18** | **9** | **8** | **8** | **9** |

**Table S13.** Effect of amino acid substitution on molecular interactions between TTR and short-chain PFAS, perfluorohexane sulfonate.

| **Perfluorohexane sulfonate** | **Wild-type TTR** | **K15G** | **L110G** | **K15G/L110G** | **K15G/L110G/**  **S117G** |
| --- | --- | --- | --- | --- | --- |
| **Binding energy** | -7.3 | -7.5 | -6.8 | -6.4 | -6.2 |
| **Number of Hydrogen bonds** | 2 | 2 | 1 | 2 | 1 |
| **Number of hydrophobic interactions** | 9 | 7 | 5 | 1 | 5 |
| **Total interactions** | **11** | **9** | **6** | **3** | **6** |

**Table S14.** Effect of amino acid substitution on molecular interactions between TTR and short-chain PFAS, perfluoroethane sulfonic acid.

| **Perfluoroethane sulfonic acid** | **Wild-type TTR** | **K15G** | **L110G** | **K15G/L110G** | **K15G/L110G/**  **S117G** |
| --- | --- | --- | --- | --- | --- |
| **Binding energy** | -4.9 | -5.2 | -4.8 | -4.7 | -4.5 |
| **Number of Hydrogen bonds** | 1 | 4 | 4 | 4 | 4 |
| **Number of hydrophobic interactions** | 6 | 3 | 2 | 3 | 2 |
| **Total interactions** | **7** | **7** | **6** | **7** | **6** |

**Table S15.** Effect of amino acid substitution on molecular interactions between TTR and short-chain PFAS, trifluoromethane sulfonic acid.

| **Trifluoromethane**  **sulfonic acid** | **Wild-type**  **TTR** | **K15G** | **L110G** | **K15G/L110G** | **K15G/L110G/**  **S117G** |
| --- | --- | --- | --- | --- | --- |
| **Binding energy** | -4.2 | -4.4 | -4 | -4 | -3.9 |
| **Number of Hydrogen**  **bonds** | 5 | 4 | 5 | 5 | 2 |
| **Number of hydrophobic**  **interactions** | 9 | 3 | 2 | 2 | 2 |
| **Total interactions** | **14** | **7** | **7** | **7** | **4** |

**Table S16.** Effect of amino acid substitution on molecular interactions between TTR and short-chain PFAS, trifluoroacetic acid.

| **Trifluoroacetic acid** | **Wild-type**  **TTR** | **K15G** | **L110G** | **K15G/L110G** | **K15G/L110G/**  **S117G** |
| --- | --- | --- | --- | --- | --- |
| **Binding energy** | -4 | -4.1 | -3.7 | -3.6 | -3.4 |
| **Number of Hydrogen**  **Bonds** | 6 | 1 | 1 | 3 | 1 |
| **Number of hydrophobic**  **interactions** | 11 | 5 | 5 | 3 | 5 |
| **Total interactions** | **17** | **6** | **6** | **6** | **6** |

**Table S17.** Comparison of the binding energy of PFASs with PPARγ ([Almeida et al. 2021](#_ENREF_1)) vs. TTR protein (data from our study).

| **(A) Long-chain PFASs** | **Binding energy with PPARγ (kcal/mol)** | **Binding energy with TTR (kcal/mol)** |
| --- | --- | --- |
| Perfluorotetradecanoic acid (PFTeDA) | -2.5 | -9.8 |
| Perfluorodecanoic acid (PFDA) | -1.3 | -8.7 |
| Perfluoroundecanoic acid (PFUnDA) | -8.2 | -9.3 |
| Perfluorodecane sulfonic acid (PFDS) | -8.8 | -9.1 |
| Perfluorodecanoic acid (PFDA) | -1.3 | -8.7 |
| Perfluorononanoic acid (PFNA) | -8.2 | -8.5 |
| Perfluorooctane sulfonic acid (PFOS) | -9.9 | -8.2 |
| Perfluorooctanoic acid (PFOA) | -6.6 | -8 |
| Perfluoroheptanoic acid (PFHpA) | -8.9 | -7.6 |
| 6:2 fluorotelomer alcohol (6:2 FTOH) | -9.2 | -7.5 |
| Perfluorododecanoic acid (PFDoA) | 3.5 | -9.4 |
| **(B) Short-chain PFASs** | **Binding energy with PPARγ (kcal/mol)** | **Binding energy with TTR (kcal/mol)** |
| Perfluorohexanoic acid (PFHxA) | -7.4 | -7.4 |
| Perfluorobutane sulfonic acid (PFBS) | -18.1 | -6.9 |
| Perfluoropentanoic acid (PFPeA) | -12.8 | -6.8 |
| Perfluorobutanoic acid (PFBA) | -17.8 | -6 |

**Table S18.** Single amino acid polymorphisms/substitution in the human population and their clinical significance

| **Sl.no.** | **Mutant/Variant** | **Clinical significance** | **Reference** |
| --- | --- | --- | --- |
| 1 | A108R | Not provided | ([Cao et al. 2020](#_ENREF_2)) |
| 2 | A108T | Amyloidogenic transthyretin amyloidosis | ([ClinVar-NCBI 2022](#_ENREF_3)) |
| 3 | A108Y/L110E | Less prone to aggregate into amyloid fibrils than wild-type TTR | ([Hörnberg 2004](#_ENREF_5)) |
| 4 | A109T | Benign and cardiovascular phenotype | ([GeneReviews-NCBI 2022](#_ENREF_4); [Skrahina et al. 2021](#_ENREF_6)), |
| 5 | A109V | Benign | ([GeneReviews-NCBI 2022](#_ENREF_4); [Skrahina et al. 2021](#_ENREF_6)) |
| 6 | A109S | Peripheral neuropathy and  autonomic neuropathy | ([GeneReviews-NCBI 2022](#_ENREF_4); [Skrahina et al. 2021](#_ENREF_6)) |
| 7 | L110M | Not provided | ([Cao et al. 2020](#_ENREF_2)) |
| 8 | L110R | Not provided | ([Cao et al. 2020](#_ENREF_2)) |
| 9 | T119M | Non-amyloid, familial euthyroid hypertyroxinemia | ([GeneReviews-NCBI 2022](#_ENREF_4); [Skrahina et al. 2021](#_ENREF_6)) |

**Reference**

Almeida NM, Eken Yi, Wilson AK (2021) Binding of Per-and Polyfluoro-alkyl Substances to Peroxisome Proliferator-Activated Receptor Gamma. ACS omega 6(23):15103-15114. doi:https://doi.org/10.1021/acsomega.1c01304

Cao Q, Anderson DH, Liang WY, Chou J, Saelices L (2020) The inhibition of cellular toxicity of amyloid-beta by dissociated transthyretin. J Biol Chem 295(41):14015-14024. doi:10.1074/jbc.RA120.013440

ClinVar-NCBI (2022) https://[www.ncbi.nlm.nih.gov/clinvar/?gr=1&term=ttr%5Bgene%5D&redir=gene](http://www.ncbi.nlm.nih.gov/clinvar/?gr=1&term=ttr%5Bgene%5D&redir=gene).

GeneReviews-NCBI (2022) https://[www.ncbi.nlm.nih.gov/books/NBK1194/table/tfap.T.ttr_variants_discussed_in_this_ge/#_ncbi_dlg_citbx_NBK1194](http://www.ncbi.nlm.nih.gov/books/NBK1194/table/tfap.T.ttr_variants_discussed_in_this_ge/#_ncbi_dlg_citbx_NBK1194).

Hörnberg A (2004) Transthyretin from a structural perspective. Umeå centrum för molekylär patogenes (UCMP)(Teknisk-naturvetenskaplig fakultet)

Skrahina V, Grittner U, Beetz C, et al. (2021) Hereditary transthyretin-related amyloidosis is frequent in polyneuropathy and cardiomyopathy of no obvious aetiology. Ann Med 53(1):1787-1796. doi:10.1080/07853890.2021.1988696
